# Proposition of Adaptive Read Bias: A Solution to Overcome Power and Scaling Limitations in Ferroelectric‐Based Neuromorphic System

**DOI:** 10.1002/advs.202303735

**Published:** 2023-12-01

**Authors:** Ryun‐Han Koo, Wonjun Shin, Seungwhan Kim, Jiseong Im, Sung‐Ho Park, Jong Hyun Ko, Dongseok Kwon, Jae‐Joon Kim, Daewoong Kwon, Jong‐Ho Lee

**Affiliations:** ^1^ Inter‐University Semiconductor Research Center Department of Electrical and Computer Engineering Seoul National University Seoul 08826 South Korea; ^2^ Department of Electrical Engineering Hanyang University Seoul 04763 South Korea; ^3^ Ministry of Science and ICT Sejong 30109 South Korea

**Keywords:** ferroelectric, hafnium oxide, low‐frequency noise, neuromorphic, power‐efficient

## Abstract

Hardware neuromorphic systems are crucial for the energy‐efficient processing of massive amounts of data. Among various candidates, hafnium oxide ferroelectric tunnel junctions (FTJs) are highly promising for artificial synaptic devices. However, FTJs exhibit non‐ideal characteristics that introduce variations in synaptic weights, presenting a considerable challenge in achieving high‐performance neuromorphic systems. The primary objective of this study is to analyze the origin and impact of these variations in neuromorphic systems. The analysis reveals that the major bottleneck in achieving a high‐performance neuromorphic system is the dynamic variation, primarily caused by the intrinsic 1/*f* noise of the device. As the device area is reduced and the read bias (*V*_Read_) is lowered, the intrinsic noise of the FTJs increases, presenting an inherent limitation for implementing area‐ and power‐efficient neuromorphic systems. To overcome this limitation, an adaptive read‐biasing (ARB) scheme is proposed that applies a different *V*_Read_ to each layer of the neuromorphic system. By exploiting the different noise sensitivities of each layer, the ARB method demonstrates significant power savings of 61.3% and a scaling effect of 91.9% compared with conventional biasing methods. These findings contribute significantly to the development of more accurate, efficient, and scalable neuromorphic systems.

## Graphical abstract

A novel biasing scheme called adaptive read bias (ARB) is proposed to address the inherent trade‐offs between power efficiency and scalability in neuromorphic systems using ferroelectric tunnel junctions (FTJs). By exploiting different noise sensitivities in each neural network, this approach achieves a 61.3% reduction in power consumption and a 91.9% improvement in scalability, representing breakthrough advances for neuromorphic computing systems.

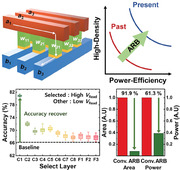

## 1 Introduction

The von Neumann architecture, which has served as the foundation of computing architecture for decades, is countering challenges owing to the increasing prevalence of data‐centric cognitive tasks such as computer vision, natural language processing, and autonomous driving.^[^ ^1^, ^2^, ^3^ ^]^ These data‐centric cognitive tasks require frequent and inefficient transfers between the processor and memory, which consume significant time and power. Neuromorphic computing, which mimics the structure of the human brain, is considered a promising solution.^[^ ^1^, ^2^, ^3^, ^4^, ^5^, ^6^ ^]^ The human brain contains many synapses that interact to compute and store data simultaneously. Therefore, for the successful implementation of neuromorphic systems, the appropriate physical devices must be selected to mimic biological synapses.

Numerous device candidates are available for the implementation of neuromorphic systems. The key difference between them is the representation of the degree of activation of the biological synaptic weights. For instance, resistive random‐access memory (ReRAM) stores information as a resistance,^[^ ^7^, ^8^, ^9^, ^10^ ^]^ phase‐change memory (PCM) stores it as a phase change,^[^ ^11^, ^12^, ^13^, ^14^ ^]^ magnetic tunnel junction (MTJ) stores it magnetically,^[^ ^15^, ^16^, ^17^, ^18^ ^]^ and ferroelectric (FE)‐based memory stores it in a polarization state.^[^ ^19^, ^20^, ^21^, ^22^, ^23^ ^]^ To realize highly integrated, low‐power, high‐performance neuromorphic systems, synaptic devices require high integration, low current density, high reliability, and high operating speed. However, PCM and MTJ suffer from high power consumption owing to their high current density, and ReRAMs suffer from reliability issues when learning frequently.^[^ ^10^, ^14^, ^18^ ^]^ In contrast, ferroelectric tunnel junctions (FTJs), which have the advantages of fast operation speed, low current density, high reliability, process simplicity, and high integration (4*F* ^2^, *F*: Minimum feature size), are considered the strongest candidates for implementing synapses in neuromorphic computing systems.^[^ ^20^, ^21^, ^22^, ^23^, ^24^, ^25^ ^]^ This movement gained significant traction following the breakthrough discovery of ferroelectricity in hafnium oxide (HfO_2_) thin films in 2011, which was fully compatible with conventional CMOS processes. Subsequently, numerous studies have reported the possibility of implementing neuromorphic systems using HfO_2_‐FTJs.^[^ ^22^, ^23^, ^24^, ^25^ ^]^

To implement neuromorphic systems based on electrical devices such as HfO_2_‐FTJ, reliability is a crucial challenge that limits the performance and accuracy of large‐scale arrays. Therefore, several studies have analyzed the impact of device reliability, such as nonlinearity, asymmetry, dynamic range, endurance, retention, read/write disturbances, and variations in neuromorphic systems.^[^ ^26^, ^27^, ^28^, ^29^ ^]^ Among these, variation becomes critical as neuromorphic systems are integrated at large scales. This can be analyzed from three perspectives. First, at the single‐device level, implementing multiple synaptic conductances reduces the number of synaptic devices used in the system and enhances network performance. However, device variation makes it difficult to distinguish between different conductances.^[^ ^30^, ^31^, ^32^ ^]^ Second, at the system level, the human brain has more than 10^15^ synapses, and neuromorphic systems that imitate them increase the number of synapses exponentially as the amount of data to be addressed increases. Because it is impossible for such a large number of synapses to have the same electrical properties, system‐level variation is introduced, which is a critical issue for large‐array operations in practice.^[^ ^33^, ^34^, ^35^ ^]^ Finally, to achieve highly integrated neuromorphic systems, the synaptic elements are gradually scaled down, leading to increased variation at both the single‐device and system levels. Therefore, it is necessary to properly evaluate the impact of reliability issues, particularly variations, as we move toward the implementation and miniaturization of neuromorphic systems.^[^ ^36^, ^37^ ^]^

**Figure 1:**
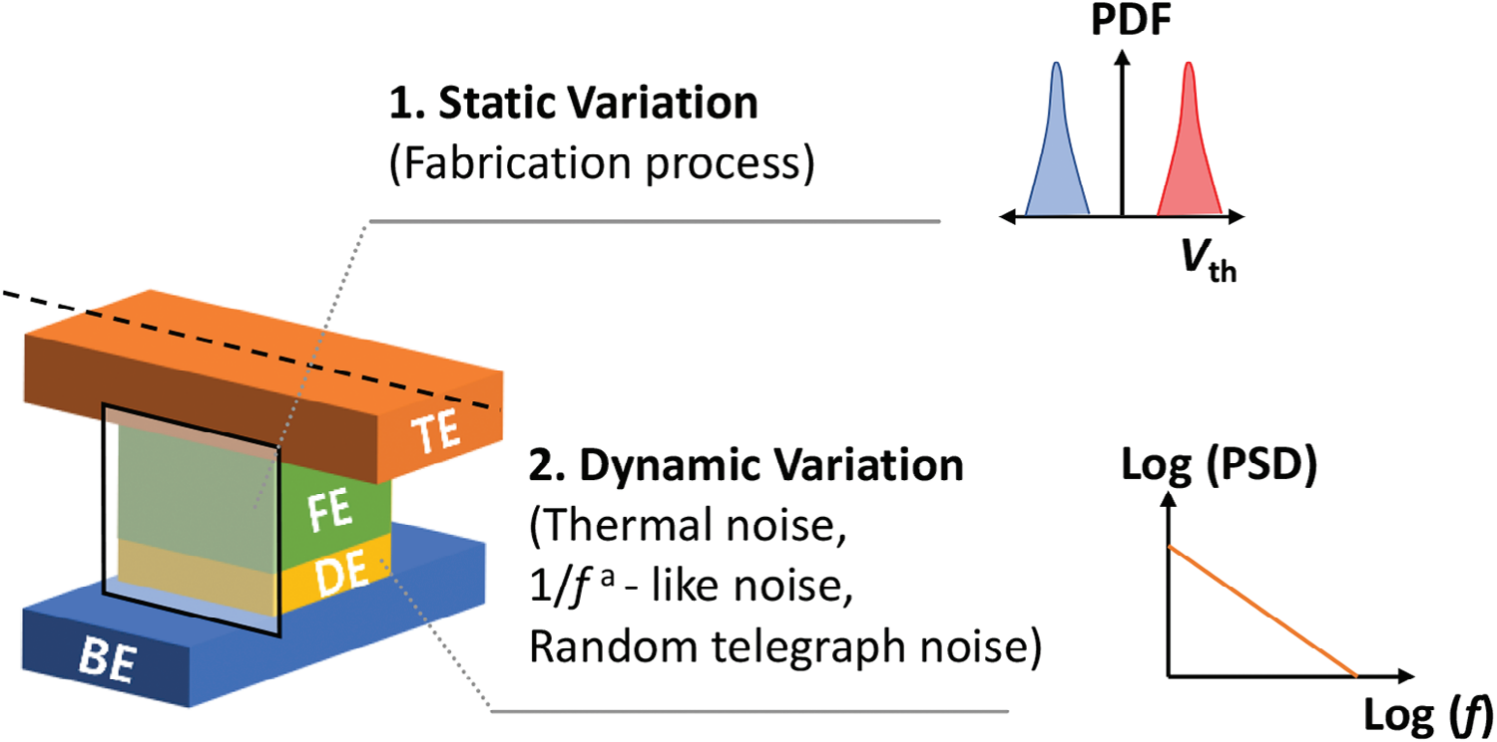
Schematic diagram of the variation in the HfO_2_‐FTJ variation sources. a) Static variation, which remains constant over time. The device fabrication process induced device‐to‐device static variation. Inset shows a schematic diagram of the probability density function (PDF) versus threshold voltage (*V*_th_) of different memory states. b) Dynamic variation, which changes continuously over time. Device‐intrinsic read noise, such as thermal noise, 1/*f*‐like noise, and random telegraph noise (RTN), is the source of dynamic variation. The inset shows a schematic diagram of power spectral density (PSD) versus frequency (*f*).

To understand how variations affect neuromorphic systems, a deeper understanding of the types of device variations is required. Here, it is imperative to differentiate between variation, which encompasses random fluctuations in device parameters, and cycle‐induced degradation or retention degradation, which are non‐random, directional phenomena with distinct causes and effects. This distinction is made because the essence of variation lies in the random distribution of a particular parameter. In contrast, cycle‐induced variation or retention degradation does not exhibit randomness; instead, it follows a specific degradation pattern. For instance, retention characteristics degrade in a specific direction, where the memory window narrows or closes. Additionally, cycle‐induced degradation leads to the collapse of the memory window, which is not a random occurrence but rather a deterministic phenomenon with a clear cause‐and‐effect relationship. **Figure** **1** shows the different sources of variation in the HfO_2_‐FTJs. These variations are divided into two categories based on their behavior over time. Static variation maintains a constant origin that remains consistent over time, whereas the source of dynamic variation undergoes continuous changes as time progresses. During device fabrication, various factors such as SiO_2_ and HfO_2_ film thickness, trap density, and size of ferroelectric domains in the FE layer vary.^[^ ^25^, ^38^, ^39^ ^]^ These are expressed as device‐to‐device variations, classified as static variations, in that they do not change over time. However, there is variation due to device intrinsic read noise, such as thermal noise, 1/*f*‐like noise, and random telegraph noise (RTN), which is classified as dynamic noise because it changes over time.^[^ ^40^, ^41^, ^42^, ^43^, ^44^, ^45^, ^46^, ^47^, ^48^, ^49^ ^]^

Because different neuromorphic system structures have different sensitivities to each variation, careful consideration of the system architecture is required to evaluate the impact of these variations on neuromorphic systems.^[^ ^29^ ^]^ Neuromorphic systems can be classified into two categories based on their training methods. In off‐chip training, the software‐trained weight map is pre‐encoded to the conductance of the physical devices.^[^ ^50^, ^51^ ^]^ In contrast, on‐chip training is based on real‐time system‐level conductance adjustment by calculating the error between the target value and the vector‐matrix multiplication (VMM) result.^[^ ^51^, ^52^ ^]^ These two methods have fundamental differences in their learning processes; therefore, the effects of these variations are also different. From the above discussion, the performance of neuromorphic systems must be re‐evaluated in different training scenarios (off‐chip and on‐chip), considering their static and dynamic variations, as well as other non‐idealities of physical devices. Furthermore, it is necessary to propose methods that reduce variations at the device level and minimize their impact at the system level, as highly integrated hardware neuromorphic systems are being developed on a large scale.^[^ ^30^, ^31^, ^32^, ^33^, ^34^, ^35^, ^36^, ^37^ ^]^

In this study, we examine the effects of static and dynamic variations on a hardware neuromorphic system based on HfO_2_‐FTJs. A 32 × 24 HfO_2_‐FTJ array is fabricated to analyze the static and dynamic variations. In addition to the electrical characteristics of the device, low‐frequency noise (LFN) spectroscopy is used to understand the causes of variation. By rigorously studying the origin of the LFN of the HfO_2_‐FTJs, we propose an innovative approach to mitigate the dynamic variation at the device level: Applying a high read bias (*V*_Read_) to the device. This reduces the dynamic variation, which increases with device downsizing, potentially overcoming the scaling limit of FTJs. However, at the system level, increasing *V*_Read_ leads to higher power consumption, resulting in a trade‐off between power consumption and synaptic density. To address this issue, we propose a novel biasing method termed “adaptive read bias” (ARB) to mitigate the impact of dynamic variation at the system level. By utilizing the ARB method, we achieve a 61.3% reduction in power consumption and a 91.9% scaling effect without compromising the accuracy of the overall neuromorphic system. Therefore, the ARB method provides a general solution for implementing power‐ and area‐efficient neuromorphic systems.

## 2 Results and Discussion

### 2.1 Electrical Characteristics of the TiN/HfO_2_/SiO_2_/*n* ^+^‐Si FTJ

**Figure 2:**
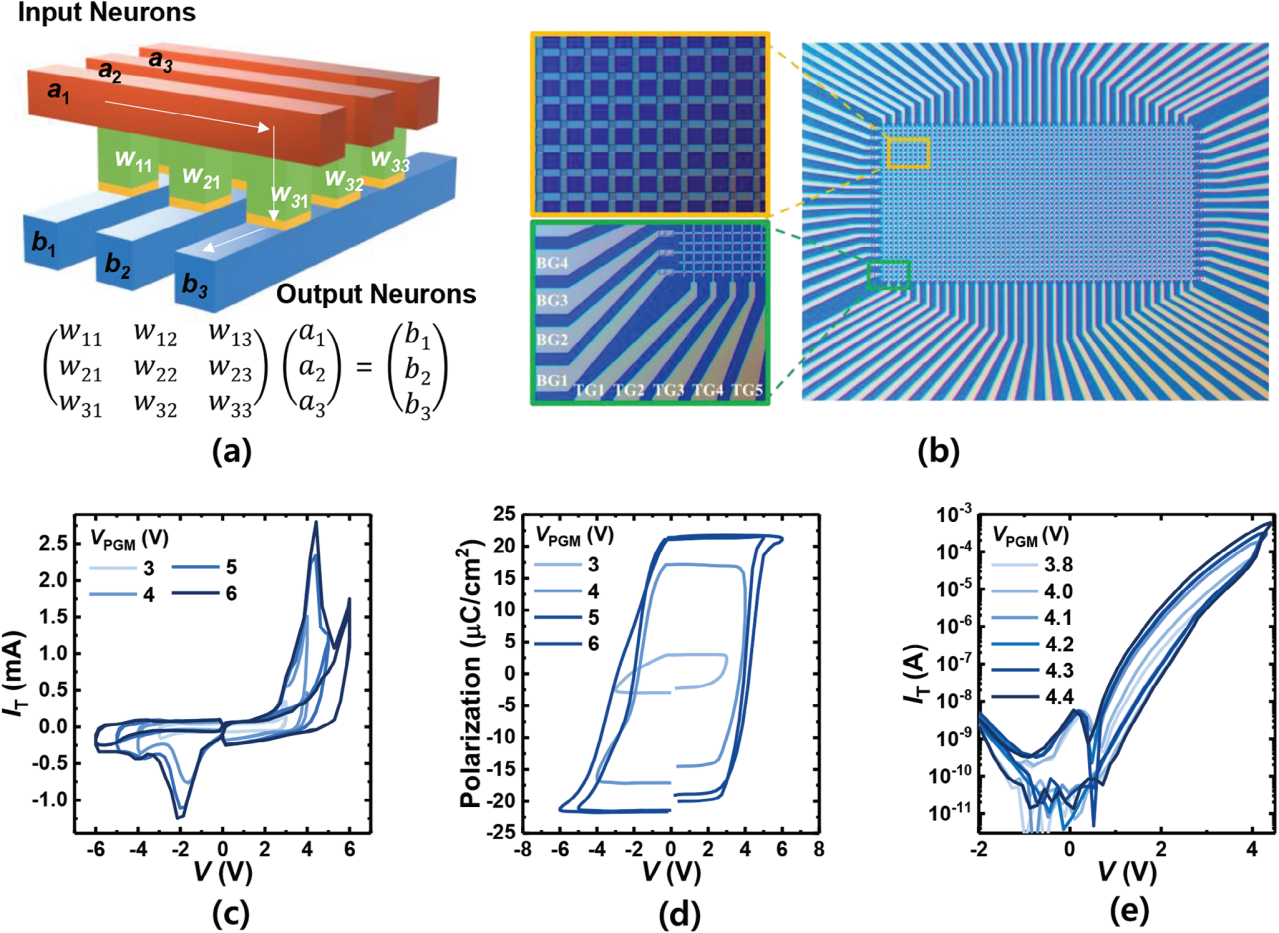
Electrical characteristics of HfO_2_‐FTJ. a) Schematic diagram of the matrix array for a neuromorphic system based on HfO_2_‐FTJ. b) Optical microscopic image of the 36 × 24 HfO_2_‐FTJ matrix array. c) PUND measurement results with different *V*_PGM_. For PUND measurement, 10 µs ramp time, and 1 µs hold time is used. d) Polarization versus *V* as a parameter of *V*_PGM_. e) Double sweep *I*_T_ versus *V* as a parameter of *V*_PGM_.

**Figure** **2**a shows a schematic of the synaptic array used for neuromorphic computing. The crossbar array of the FTJ can be used as the matrix component of an artificial neural network consisting of a two‐terminal artificial synapse connecting the pre‐ and post‐synaptic neurons. The FTJ array mimics synaptic transmission by multiplying the input neuron signal (encoded as the voltage applied to the device) by the corresponding weight (conductance) and passing the multiplication product (resulting current) to the output neuron. Dense, fast, and power‐efficient VMM computation is thus inherently possible at the physical level using Ohm's and Kirchoff's laws.^[^ ^1^, ^2^, ^3^, ^4^, ^5^, ^6^ ^]^ Figure 2b shows an optical microscopy image of a 32 × 24 crossbar array with HfO_2_‐based FTJ nodes. The FTJ cells are positioned at the intersections of perpendicular Ti/TiN/Al/TiN electrode lines. The fabrication process is illustrated in Figure S1, Supporting Information. Figure S2, Supporting Information shows a cross‐sectional transmission electron microscopy (TEM) image of the (metal‐ferroelectric‐insulator‐semiconductor) MFIS HfO_2_‐FTJ, where the thicknesses of the TiN, HfO_2,_ and SiO_2_ are 100, 6, and 1.2 nm, respectively. Table S1, Supporting Information shows the area of the fabricated device, the area where power spectral density (PSD) measurements are performed, and the area of the theoretically studied device.

To emulate the learning mechanisms of the biological brain effectively, a hardware neuromorphic system requires additional functionality beyond the VMM operations mentioned above. The postsynaptic current of each device cell should be gradually modified based on the history of the applied pulse spikes from the presynaptic neuron, enabling learning and memory functions.^[^ ^12^, ^53^ ^]^ To achieve a gradual conductance change (multiple conductance levels) in response to input spikes, FE‐based devices exploit the partial polarization of the FE layer. In Figure 2c, the positive‐up‐negative‐down (PUND) measurement results of the FTJ demonstrate the existence of ferroelectric switching in the fabricated HfO_2_ layer owing to the polarization current. Figure 2d shows the relationship between the polarization (*P*) and voltage applied to the top gate (*V*) as a parameter of the maximum program voltage (*V*_PGM_). As *V*_PGM_ increases, the remnant polarization (*P*_r_) also increases, indicating partial polarization property.^[^ ^54^ ^]^ Figure 2e shows the DC double‐sweep tunneling current (*I*_T_) versus *V* as a function of *V*_PGM_. Because of the partial polarization of the FE layer, the tunneling electroresistance (TER) ratio increased with increasing *V*_PGM_. These results show that when an input voltage is applied to synaptic devices for learning, not all domains of the ferroelectric layer are switched at once, but rather, they are switched in part, demonstrating that multiple conductance levels can be achieved for neuromorphic devices.

**Figure 3:**
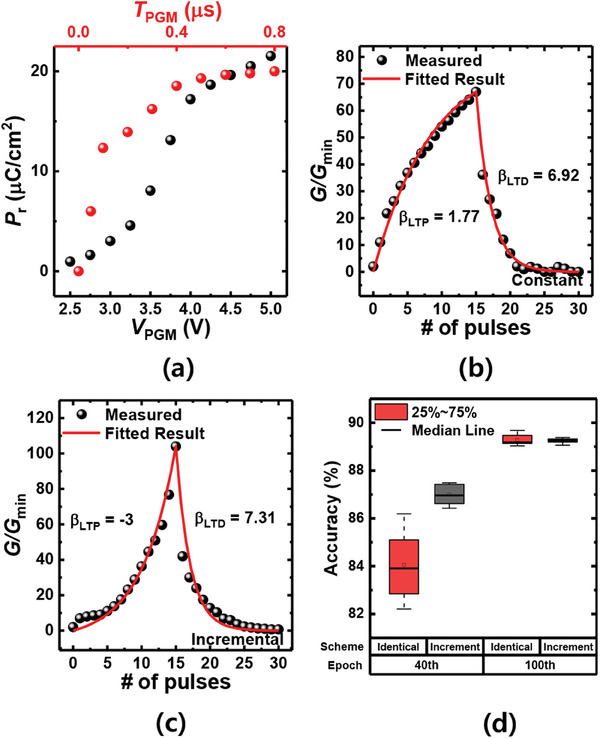
Synaptic characteristics of HfO_2_‐FTJ. a) *P*_r_ versus *V*_PGM_ (black symbol) and *T*_PGM_ (red symbol). For the *P*_r_ versus *V*_PGM_ measurement, *T*_PGM_ is fixed at 1 µs. For the *P*_r_ versus *T*_PGM_, *V*_PGM_ is fixed at 5 V. b) LTP‐LTD characteristics of HfO_2_‐FTJ with an identical pulse scheme. An identical pulse of 5 V, 1 µs is applied. The red line shows the fitting results of nonlinearity. c) LTP‐LTD characteristics of HfO_2_‐FTJ with incremental pulse scheme. Program pulses with increasing amplitude of 4.0 to 5.5 V, 1 µs are applied. The red line shows the fitting result of the nonlinearity. d) Accuracy of the network trained with identical (red box plot) and incremental pulse schemes (blue box plot) for the 40^th^ and 100^th^ epochs.

In addition to multiple conductances, nonlinearity plays a critical role in the implementation of high‐performance neuromorphic systems.^[^ ^29^ ^]^ Nonlinearity determines the rate at which the conductance changes with the number of voltage inputs, thereby affecting the training accuracy. If nonlinearity is significant, the updated value may deviate from the intended value, resulting in detrimental effects on the learning speed and accuracy of the neuromorphic system. In FE‐based devices, nonlinearity depends on how the input voltage pulse is applied to update the conductance; therefore, we experiment with two schemes: Identical and incremental pulse schemes. **Figure** **3**a shows the dependence of *P*_r_ on *V*_PGM_ (black symbols) and *T*_PGM_ (red symbols). The change in *P*_r_ with *V*_PGM_ is related to the conductance change in the incremental pulse scheme and the *P*_r_ change with *T*_PGM_ is related to the constant pulse scheme. In general, the constant pulse scheme is easier to implement, whereas the incremental pulse scheme provides higher accuracy.^[^ ^55^, ^56^, ^57^ ^]^ Figures 3b,c show the behavior of long‐term potentiation (LTP) and long‐term depression (LTD) using identical and incremental pulse schemes, respectively. Figure S3, Supporting Information shows *P*–*V*, endurance, retention, and LTP‐LTD characteristics of the HfO_2_‐FTJ array. Figure S4, Supporting Information shows the programming operation scheme in the HfO_2_‐FTJ array. The nonlinearity of each update scheme can be extracted as follows.^[^ ^29^ ^]^

(1) $$W_{\mathrm{LTP}}=g_{\min}+\alpha \left(1-e^{\beta x}/N\right)$$

(2) $$W_{\mathrm{LTD}}=g_{\max}-g_{\min}-\alpha \left(1-e^{\beta x}/N\right)$$

(3) $$\alpha =\left(g_{\max}-g_{\min}\right)/\left(1-e^{-\beta }\right)$$

where *g*_max_ and *g*_min_ are the maximum and minimum conductances, respectively, and *β* is the nonlinear factor. *N* is the total number of pulses applied during the LTP (LTD). While the identical pulse scheme has a positive *β* for both LTP and LTD, the incremental pulse scheme has a negative *β* for LTP and a positive *β* for LTD. This is due to the partial polarization characteristics of the FE layer. As shown in Figures 3b,c, the first pulse in the identical pulse scheme induces a larger polarization switching compared to the incremental pulse scheme, resulting in a convex upward LTP curve with positive *β*. However, in the incremental pulse scheme, the conductance increases with *V*_PGM_, forming a downward convex LTP curve, and *β* is negative. This negative *β* in LTP is known to be a good factor for neuromorphic computing because of its symmetry with the LTD characteristics.^[^ ^56^, ^57^ ^]^ Now, we compare the learning accuracy of an identical pulse scheme and an incremental pulse scheme. Figure S4, Supporting Information shows the average learning accuracy for the ten learning curves using identical and incremental pulse schemes. In both cases, the learning accuracy increased with some fluctuations in the epoch. The cause of this fluctuation is that the intended weight update value is distorted by the nonlinearity of the synaptic devices.^[^ ^29^ ^]^ In the case of an incremental pulse, the scheme exhibits less fluctuation and faster convergence. This is because the mismatch between LTP and LTD is less than that in an identical pulse scheme. Figure 3d shows the accuracy of the identical (red box plot) and incremental (blue box plot) pulse schemes for the 40^th^ (early stage) and 100^th^ (late stage) epochs. The learning curves are shown in Figure S5, Supporting Information. As expected, the accuracy of the 40^th^ epoch with the incremental pulse scheme is higher than that of the identical pulse scheme owing to the fast convergence speed with symmetric LTP‐LTD characteristics. However, in the 100^th^ epoch, the identical pulse scheme fully converged, and there was no difference in the final accuracy. Although a difference occurred in the convergence speed, we believe that the identical pulse method is suitable for on‐chip learning because there is no significant difference in the final accuracy, and the hardware and software costs are very low compared to those of the incremental pulse method. To implement a neuromorphic system using an incremental pulse scheme, it is necessary to store the historical data of the applied pulses in each device, which requires significant memory and computing power. This becomes even more challenging considering that even a light MobileNet model consists of over one million parameters.^[^ ^58^, ^59^, ^60^ ^]^ Consequently, employing an incremental pulse scheme in real‐world applications is not feasible. Consequently, this study relies on simulations using an identical pulse scheme to examine the impact of the variations.

### 2.2 Origin of HfO_2_‐based FTJ Variation and Its Impact on the Hardware Neuromorphic System

**Figure 4:**
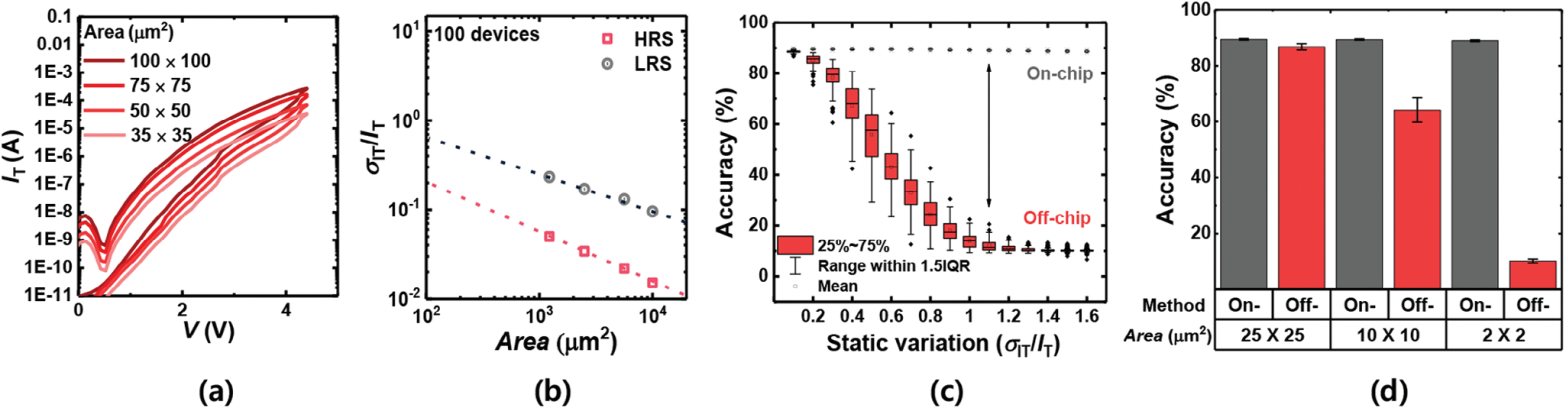
Impact of the static variation on the hardware neuromorphic system. a) Double sweep *I*_T_ versus *V* as a parameter of area. b) Correlation between the area and normalized current variation (*σ*_IT_/*I*_T_) of 100 HfO_2_‐FTJs in LRS and HRS. c) Accuracy after performing on‐chip and off‐chip learning in the presence of static variation. d) Final accuracy of the network with respect to learning method and area of the devices.

In addition to nonlinearity and asymmetry, we analyze the effect of variations (static and dynamic variations) on hardware neuromorphic systems depending on the learning methods (on‐chip and off‐chip learning methods). Figure S6, Supporting Information shows a schematic diagram of the simulation methodology. First, we analyze the effects of static variation on the neuromorphic system. We measured 100 devices with areas of 100 × 100, 75 × 75, 50 × 50, and 35 × 35 µm^2^. **Figure** **4**a shows the DC double‐sweep *I*_T_ versus *V* as a function of the area. As the area is scaled down, *I*_T_ decreases because the current density remains constant. Notably, the FTJ has a very low current density compared with other 2‐terminal memories (ReRAM, PCM, and MTJ), highlighting its potential for extremely low‐power operation of hardware neuromorphic systems. Figure 4b shows the correlation between the area and the normalized current variation (*σ*_IT_/*I*_T_) of the FTJs in the low‐resistive state (LRS) and high‐resistive state (HRS). As the device is scaled down, the fabrication‐induced variation increases because variables such as the interface trap density, HfO_2_ film thickness, and number of domains become less uniform.^[^ ^36^, ^37^ ^]^ These fabrication process‐induced variations are categorized as static because their origins are established during the fabrication process itself. Depending on how the neuromorphic system is trained, its sensitivity to static variation also varies; therefore, we investigate the effect of static variation using on‐chip and off‐chip training methods. Figure 4c show the accuracy after off‐chip and on‐chip training versus the static variation. The results shows that static variation degraded the accuracy of the off‐chip training method, whereas the accuracy of the on‐chip training remained constant. This significant difference arises from the consideration of static variations during training. In off‐chip learning, the weight map trained by the software is pre‐encoded to the conductance of physical devices. Therefore, if a static variation exists, the parameter weights learned from the external computer are not correctly transferred, resulting in accuracy degradation. In contrast, on‐chip training is based on a real‐time system‐level conductance adjustment by calculating the error between the target value and the VMM result. Therefore, unlike off‐chip training, on‐chip training is robust to this static variation because the training is conducted in an environment where there is already device‐to‐device variation, and the weight values are determined to account for this variation.^[^ ^29^, ^61^ ^]^

Figure 4d shows the results of neural network simulations with different training schemes as the FTJs are scaled down. When the off‐chip training is applied, the accuracy is maintained up to 25 × 25 µm^2^, but as the device is scaled down, the accuracy decreases. At 2 × 2 µm^2^, it returns to its initial state without training (accuracy ≈ 10%). However, in the case of on‐chip training, there is no decrease in accuracy as the device is scaled down. In addition, on‐chip training, which updates the weight and performs VMM computation on the same hardware platform, does not require extracting or encoding the weight between the software and hardware, ultimately improving the area/energy efficiency.^[^ ^50^, ^51^ ^]^ Therefore, on‐chip training is essential for implementing size‐ and power‐efficient neural networks with further downscaling.

**Figure 5:**
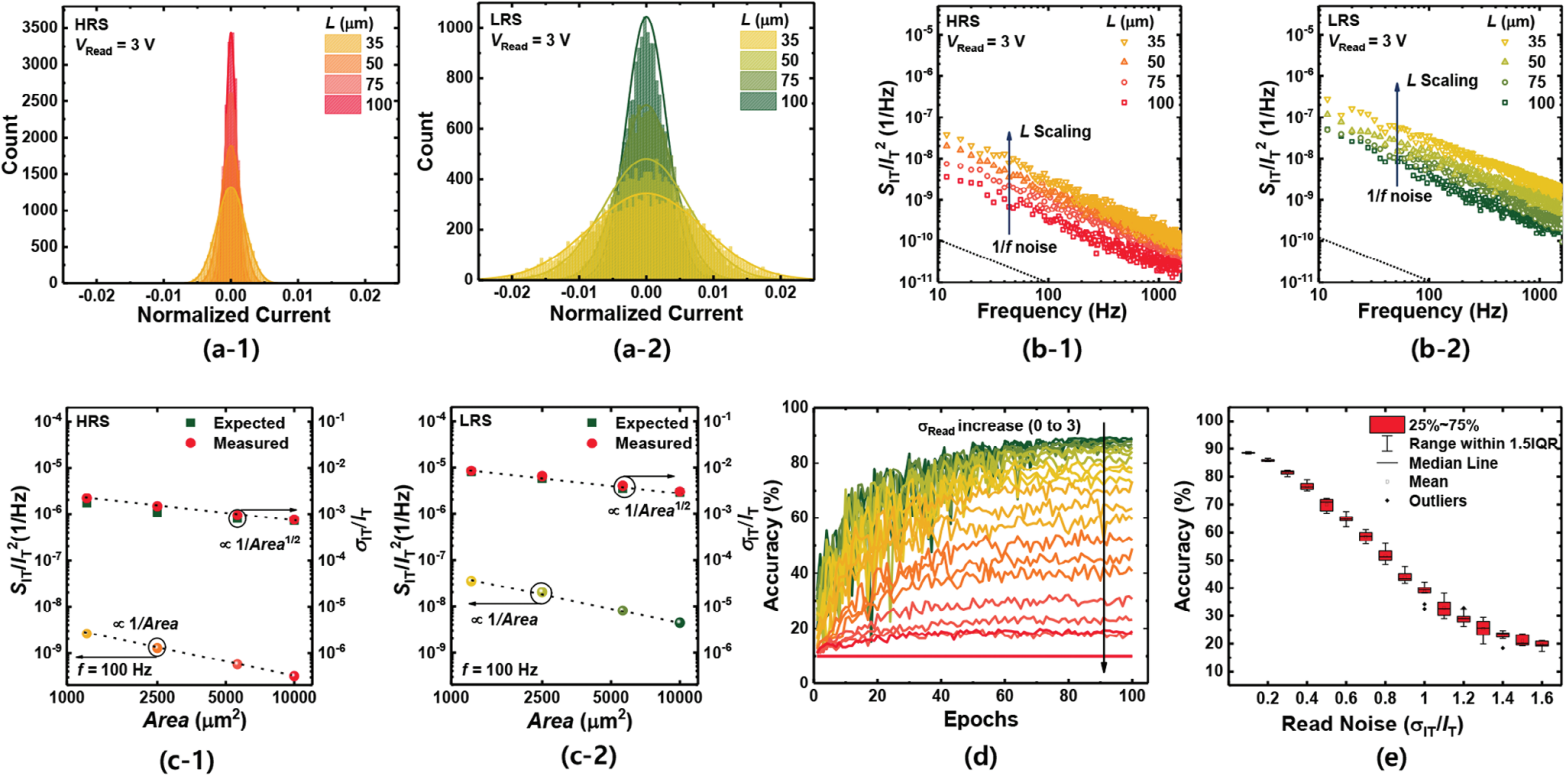
Area dependence of the dynamic variation and its impact on the hardware neuromorphic system. a) Normalized transient current counts in the HfO_2_‐FTJ in a—1) HRS and a—2) LRS. b) *S*_IT_/*I*_T_^2^ versus *f* as a parameter of the device area in b—1) HRS and b—2) LRS. c) *S*_IT_/*I*_T_^2^ (left axis) and *σ*_IT_/*I*_T_^2^ (right axis) with respect to the device area in c—1) HRS and c—2) LRS. d) Learning accuracy of the VGG‐11 network based on HfO_2_‐FTJs with identical pulse scheme as a parameter of dynamic variation (0 to 3). e) Final accuracy obtained during the learning process (10 times) with respect to *σ*_IT_/*I*_T_.

Subsequently, we analyze the impact of intrinsic device noise (dynamic variation) on an on‐chip neuromorphic system. In semiconductor devices, intrinsic device noise such as 1/*f* noise and random telegraph noise (RTN) causes dynamic variation. This is because these noise types originate from random processes generated by carrier trapping or fluctuations in barrier height, and their behavior is influenced by various defects unique to each process.^[^ ^40^, ^41^, ^42^, ^43^, ^44^, ^45^, ^46^, ^47^, ^48^, ^49^, ^62^, ^63^, ^64^, ^65^ ^]^ **Figure** **5**a shows the normalized transient *I*_T_ ($\frac{I_{T}-I_{avg}}{I_{avg}},$ *I*_avg_ is the average *I*_T_) counts in the FTJ in the a—1) HRS and a—2) LRS. In all cases, *I*_T_ follows a Gaussian distribution, and the standard deviation increases with device downscaling. To reveal the physical origin of these current fluctuations, the power spectral density (PSD) is measured for different areas (100 × 100, 75 × 75, 50 × 50, and 35 × 35 µm^2^). Figure S5, Supporting Information shows the setup for LFN spectroscopy. Figure 5b shows the normalized PSD (*S*_IT_/*I*_T_^2^) as a function of the device area in the b—1) HRS and b—2) LRS. As shown in Figure 5b, the FTJs exhibit 1/*f* ^γ^ (*γ* = − Δ*ln*(*S*)/Δ*lnf*) noise behavior in all cases. Furthermore, the *γ* value of FTJs is close to one regardless of the area. In addition, *S*_IT_/*I*_T_^2^ increases with device downscaling. By analyzing the *I*_T_–*V* characteristics at different temperatures and performing LFN spectroscopy, we demonstrate that the 1/*f* noise characteristics of FTJ in the HRS originate from shot noise at low *V*_Read_ and barrier height fluctuation (BHF) at high *V*_Read_.^[^ ^24^ ^]^ The detailed procedure is shown in Figure S8 and Text S1, Supporting Information. The shot noise owing to the direct tunneling current can be expressed as follows.^[^ ^66^, ^67^ ^]^

(4) $$S_{\mathrm{IT}}=2qFI_{T}$$

where *q* is the elementary charge and *F* is the Fano factor related to the time constant of the trapping and detrapping processes.

1/*f* noise generated from the BHF can be expressed as follows.^[^ ^68^ ^]^

(5) $$\frac{S}{I_{T}^{2}}\propto \frac{1}{fA}exp\left(\frac{qV_{bi}}{k_{B}T}\right)$$

where *A* is the area and *V*_bi_ is the built‐in potential.

By extracting the trap energy level during current conduction and LFN spectroscopy, it is revealed that the 1/*f* noise of the FTJ in the LRS was originated from the PF emission noise in all *V*_Read_. The detailed procedure is shown in Figure S9 and Text S2, Supporting Information. When 1/*f* noise is generated from the PF emission, *S*_IT_/*I*_T_^2^ is expressed as follows.^[^ ^69^ ^]^

(6) $$\frac{S}{I_{T}^{2}}=\frac{\delta^{2}N_{D}}{E\varepsilon_{ins}^{2}WL}\frac{q^{2}R}{f}$$

where *δ* denotes the fitting parameter, *N*_D_ denotes the trap density, *E* denotes the electric field, ε_*ins*_ denotes the permittivity of the insulator layer, and *R* denotes the ratio of trap time constants. In Equations (5) and (6), *S*_IT_/*I*_T_^2^ is inversely proportional to the area, so the *S*_IT_/*I*_T_^2^ increases as the device is downscaled. The left axis of Figure 5c shows the relationship between *S*_IT_/*I*_T_^2^ and area. Note that *S*_IT_/*I*_T_^2^ is sampled at 100 Hz. In both the LRS and HRS, *S*_IT_/*I*_T_^2^ is proportional to 1/*A*, which is consistent with theoretical predictions. The right axis of Figure 5c shows the relationship between the normalized standard deviation of *I*_T_ (*σ*_IT_/*I*_T_) and area. The measurement results show a 1/*A* ^1/2^ dependency. If the random fluctuation of *I*_T_ originated from the intrinsic device noise, not resistance degradation or external noise, it is possible to calculate *σ*_IT_/*I*_T_ from the plot of *S*_IT_/*I*_T_^2^ versus frequency. The *σ*_IT_ can be calculated by integrating the spectrum *S* according to the following equation.^[^ ^70^, ^71^, ^72^ ^]^

(7) $$\sigma =\int_{f_{min}}^{f_{max}}Sdf$$

where *f*_min_ = *t* ^‐1^, *f*_max_ = 2*t*_s_^‐1^, and *t*_s_ is the sampling time. We calculate the expected values *σ*_IT_/*I*_T_ using the measurement results of *S*_IT_/*I*_T_^2^ and depict them as green points in Figure 5c. In both the LRS and HRS, the expected *σ*_IT_/*I*_T_ and measured *σ*_IT_/*I*_T_ show excellent consistency, demonstrating that the random fluctuation of current originated from device intrinsic noise, not resistance degradation or external noise.^[^ ^71^ ^]^ Because a neuromorphic system utilizes currents to represent the results of VMMs, random fluctuations in the current degrade the accuracy of the neuromorphic system. In particular, device intrinsic noise, whose magnitude is increased with area scaling, may serve as a major bottleneck in the implementation of dense, power‐efficient neuromorphic systems. Figure 5d shows the learning accuracies of the VGG‐11 networks based on FTJs with identical pulse schemes as a parameter of dynamic variation. Unlike static variation, accuracy degradation due to dynamic variation cannot be recovered, even in the case of on‐chip learning. This is because, unlike static variation, dynamic variation changes with time; therefore, it cannot be compensated for during the learning process. Even if learning is performed for a specific noise direction, a completely different random noise fluctuation occurs in the next time step, such that the accuracy is saturated even if learning is fully performed. Figure 5e shows the final accuracy obtained during the learning process (10 iterations) with respect to *σ*_IT_/*I*_T_. As mentioned previously, dynamic variation is a source of accuracy degradation. We also perform a simulation of VGG‐11 networks based on 1 × 1 µm^2^ FTJs (*V*_Read_ = 2 V) using an identical pulse scheme. Thus, there is a serious accuracy degradation from 90% accuracy to 48.94% in the presence of intrinsic noise. If we use a larger device to reduce the accuracy degradation, the device should scale up to 17 × 17 µm^2^ to achieve an accuracy drop of less than 3%. Therefore, it is crucial to determine a method to reduce the intrinsic noise of a device to implement an area‐efficient hardware neuromorphic system. The key to reducing the intrinsic device noise of an FTJ is to clearly understand the noise mechanism.

**Figure 6:**
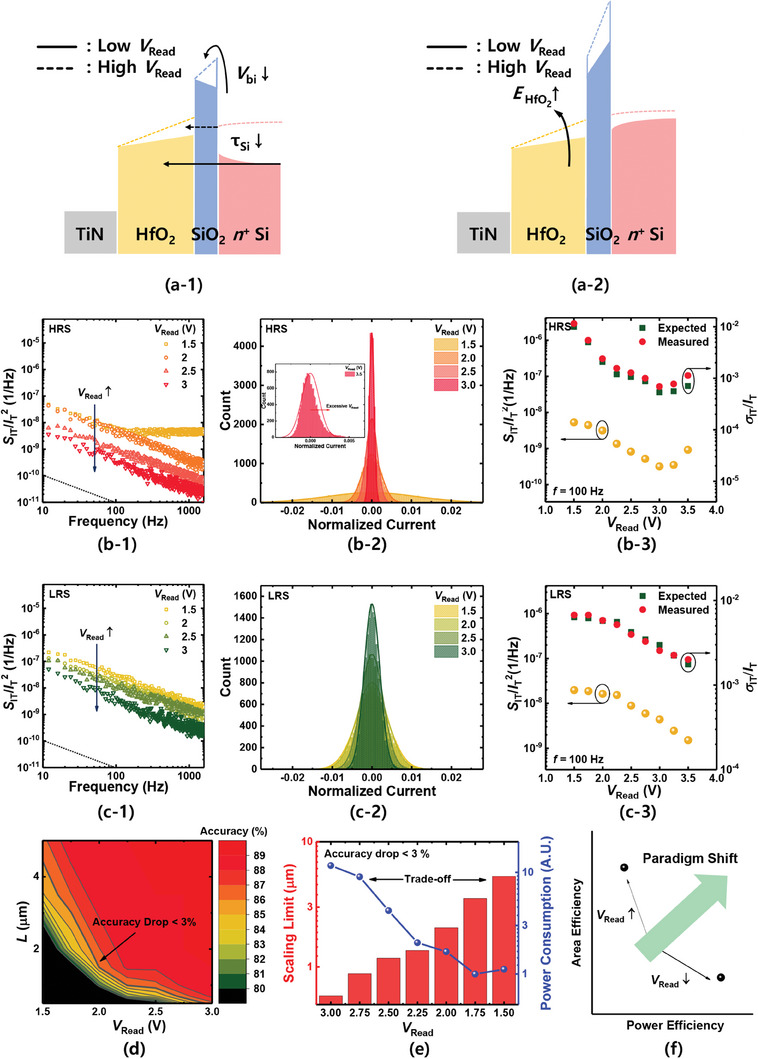
Understanding the origin of LFN in HfO_2_‐FTJ and presenting methods to reduce the dynamic variation for device downscaling. a) Schematic energy band diagram of HfO_2_‐FTJ. The dotted line indicates the application of a high *V*_Read_. b–1) *S*_IT_/*I*_T_^2^ of the FTJ in HRS and c–1) LRS as a parameter of *V*_Read_. b–2) Normalized transient *I*_T_ counts in HRS and c–2) LRS as a parameter of *V*_Read_. b–3) *S*_IT_/*I*_T_^2^ (left axis) and measured *σ*_IT_/*I*_T_ (right axis) with respect to device area in HRS and c–3) LRS. d) Relationship between HfO_2_‐FTJ area, *V*_Read_, and accuracy of the neuromorphic system. The green line indicates the condition for accuracy degradation to be less than 3%. e) Device scaling limit (red bar) and power consumption (blue line) with respect to *V*_Read_ to achieve accuracy degradation of less than 3%. f) Schematic representation of the trade‐off between area and power efficiency. The paradigm shift needs to achieve both metrics.

**Figure** **6**a shows a schematic energy band diagram of the HfO_2_‐FTJ. The dotted line indicates the band diagram obtained when a high *V*_Read_ is applied. As *V*_Read_ increased, *V*_bi_ and the time constant for electron trapping from Si (*t*_Si_) decreased in the HRS device, and the *E*‐field in the HfO_2_ layer (*E*_HfO2_) increased in the LRS device. Figure 6b–1 shows *S*_IT_/*I*_T_^2^ of the FTJ in the HRS as a function of *V*_Read_. The behavior of *S*_IT_/*I*_T_^2^ changes from shot noise to 1/*f* noise as *V*_Read_ is increased from 1.5 to 2.0 V. *τ*_Si_ is compared to the time constant for electron detrapping to TiN (*τ*_TiN_), so *τ*_Si_ determines *F*. By increasing *V*_Read_, the value of *τ*_Si_ decreases, which also decreases *F*, as shown in Figure 6b–1 (*V*_Read_ = 1.5 to 2.0 V). Furthermore, when shot noise is minimized owing to a high *V*_Read_, the presence of 1/*f* noise owing to the BHF becomes apparent.^[^ ^29^ ^]^ Thus, *S*_IT_/*I*_T_^2^ at *V*_Read_ = 2.0 V shows 1/*f* noise characteristics. When *S*_IT_/*I*_T_^2^ follows the 1/*f* noise from the BHF, the magnitude of the noise decreases as *V*_bi_ decreases with increasing *V*_Read_. Figure 6b–2 shows the normalized transient *I*_T_ counts in the FTJ of the HRS as a function of *V*_Read_. As *V*_Read_ increases, *σ*_IT_/*I*_T_ decreases, similar to the trend observed in Figure 6b–3. Note that as *V*_Read_ changes from 1.5 to 2.0 V, *σ*_IT_/*I*_T_ decreases rapidly as the LFN changes from shot noise to 1/*f* noise. The inset of Figure 6b–2 shows the normalized transient *I*_T_ counts at *V*_Read_ = 3.5 V. Unlike when *V*_Read_ ranges from 1.5 to 3.0 V, the *I*_T_ counts do not follow a Gaussian distribution, indicating that resistance degradation rather than fluctuation occurs at high *V*_Read_s.^[^ ^71^ ^]^ The left axis of Figure 6b–3 shows the sampled *S*_IT_/*I*_T_^2^ versus *V*_Read_ (yellow symbols), and the right axis shows the measured *σ*_IT_/*I*_T_. *S*_IT_/*I*_T_^2^ decreases with increasing *V*_Read_. Furthermore, *σ*_IT_/*I*_T_ decreases with increasing *V*_Read_ from 1.5 to 3.0 V. Note that σ_IT_/*I*_T_ increases with increasing *V*_Read_ above 3.0 V due to resistance degradation.

Figure 6c–1 shows the *S*_IT_/*I*_T_^2^of of the FTJ in the LRS as a function of *V*_Read_. As deduced from Equation (5), as *V*_Read_ increases, *E*_HfO2_ increases and *S*_IT_/*I*_T_^2^ decreases. In addition, unlike the LFN in the device in the HRS, *S*_IT_/*I*_T_^2^ exhibits a 1/*f* noise trend regardless of *V*_Read_. Figure 6c–2 shows the normalized transient *I*_T_ counts in the FTJ in the LRS as a function of *V*_Read_.^[^ ^29^ ^]^ Once again, as *V*_Read_ increases, *σ*_IT_/*I*_T_ decreases, as shown in Figure 6c–1. Notably, the decrease in *σ*_IT_/*I*_T_ with respect to *V*_Read_ is much slower in the LRS than in the HRS. This is due to the origin of the LFN. *S*_IT_/*I*_T_^2^ is proportional to e*^V^* ^bi^ in HRS, while *S*_IT_/*I*_T_^2^ is proportional to 1/*E*_HfO2_ in LRS. Thus, *S*_IT_/*I*_T_^2^ decreases much faster with increasing *V*_Read_ in the HRS device. The left axis of Figure 5c–3 shows the sampled *S*_IT_/*I*_T_^2^ versus *V*_Read_ (yellow symbols), and the right axis shows the measured *σ*_IT_/*I*_T_. Again, *S*_IT_/*I*_T_^2^ decreases with increasing *V*_Read_.

Consequently, we present methods to reduce the dynamic variation of the FTJ in both the HRS and LRS by applying a high *V*_Read_ bias. By increasing *V*_Read,_ the device size can be further reduced. Figure 6d shows the relationship between the FTJ area, *V*_Read_, and accuracy of the neuromorphic system. The thick green line shows the condition for reducing the accuracy to less than 3%. The red bar in Figure 6e shows the device scaling limit with respect to *V*_Read_ to achieve an accuracy degradation of less than 3%. Increasing *V*_Read_ reduces dynamic variation, allowing further device downscaling. In particular, increasing *V*_Read_ = 1.5 to 3.0 V results in an area downscaling from 5.29 × 5.29 to 0.59 × 0.59 µm^2^. The blue line and symbols in Figure 6e represent the power consumption of the neuromorphic system with respect to *V*_Read_. The power consumption and scaling limit exhibit opposite tendencies. As *V*_Read_ increases, the power consumption of the entire neuromorphic system increases if the device scaling limit improves. Notably, there is a trade‐off between the scaling limit and power consumption. Increasing *V*_Read_ allows the device to be scaled down by reducing the dynamic variation, but the power consumption increases, as shown in Figure S10, Supporting Information. Therefore, as summarized in Figure 6f, we have shown how the control of *V*_Read_ can improve either the power efficiency or area efficiency of a neuromorphic system. However, owing to the inherent trade‐off between these two attributes, it is impossible to optimize them simultaneously. This is a fundamental challenge in the realization of area‐ and power‐efficient neuromorphic systems, and novel paradigms must be explored to overcome this obstacle.

### 2.3 Demonstration of an Area‐ and Energy‐Efficient Hardware Neuromorphic System with Adaptive Read Bias

**Figure 7:**
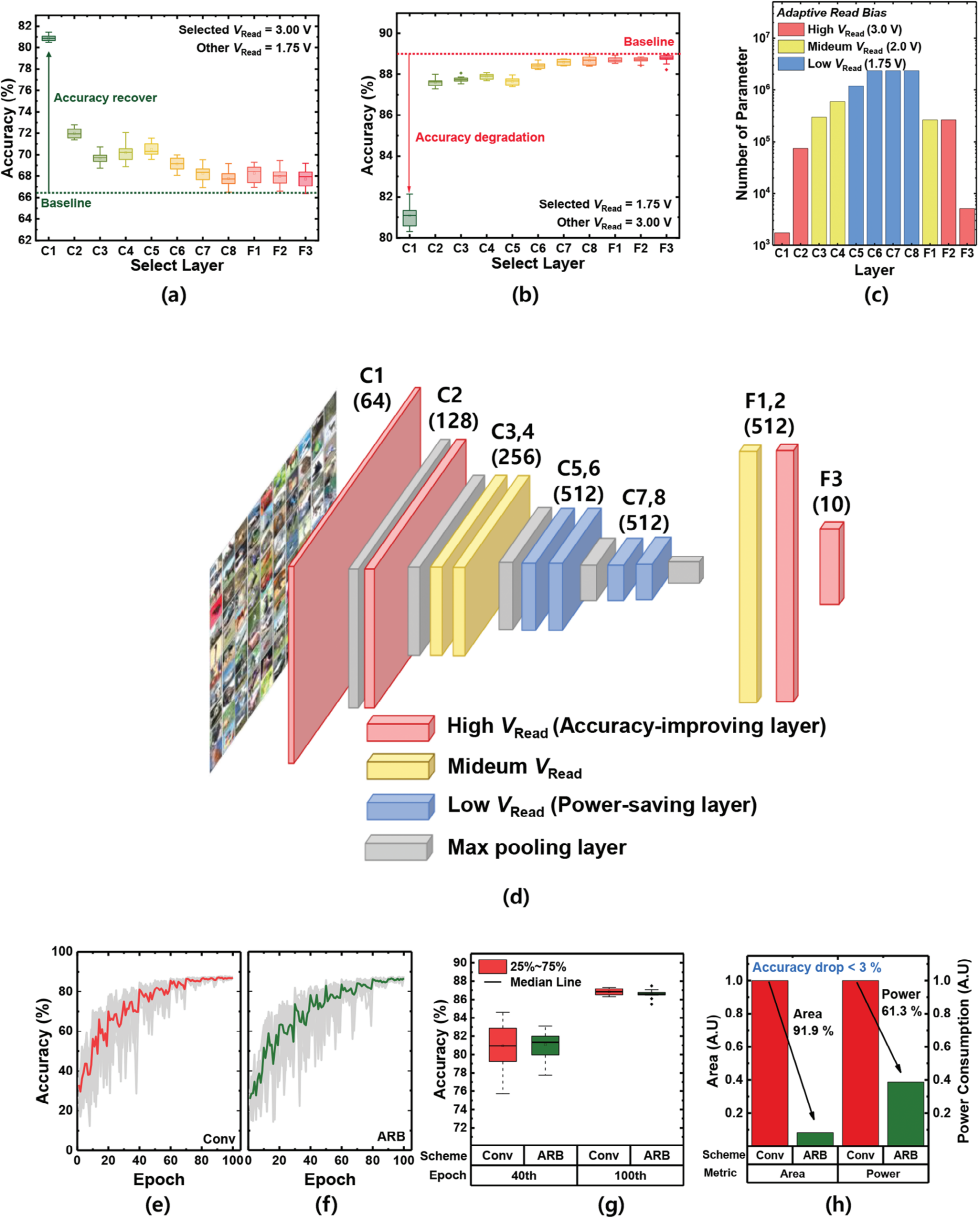
Proposed ARB methods. a) Accuracy when a selected layer is biased with a high *V*_Read_ and another layer is biased with a low *V*_Read_. b) Accuracy when the selected layer is biased with a low *V*_Read_ and other layers are biased with a high *V*_Read_. c) Number of parameters in each layer of the VGG‐11 network. d) Schematic diagram of the overall ARB methods. e) Averaged learning accuracy for the conventional biasing scheme and f) ARB scheme. g) Accuracy of the conventional biasing method (red box plot) and ARB scheme (green box plot) for the 40^th^ and 100^th^ epochs. h) Power consumption and area of the network using the conventional biasing method (red box plot) and ARB (green box plot) to achieve an accuracy degradation of less than 3%.

In the conventional neuromorphic system, the same *V*_Read_ is applied to all layers. However, previous studies reported that different layers in a neuromorphic system have different sensitivity to variation.^[^ ^73^, ^74^ ^]^ Therefore, in this paper, we propose a novel method called ARB to adopt different *V*_Read_ for each layer, enabling additional headrooms for area downscaling and power reduction. Specifically, by applying a low *V*_Read_ to the noise‐robust layer, power consumption can be reduced and area downscaling can be achieved. Conversely, applying a high *V*_Read_ to the noise‐sensitive layer enhances the accuracy of the overall system. To successfully implement ARB, it is necessary to determine the noise sensitivity of the different layers. **Figure** **7**a shows the accuracy when a selected layer is biased with a high *V*_Read_ (low read noise), whereas the other layers are biased with a low *V*_Read_ (high read noise). The green dotted line indicates the baseline accuracy when the low *V*_Read_ case is applied to all layers (66.3%). Reducing noise in the convolution (C)1 layer shows the best accuracy recovery, resulting in 15%p accuracy recovery. Furthermore, the C2 to C5 layer shows moderate accuracy recovery (5.7 to 3.4%p), whereas the C7 to the fully connected (F)3 layer shows almost no accuracy recovery (1.9%p to 1.4%p). This trend is due to the characteristics of a convolutional neural network (CNN), in which feature extraction occurs in the convolutional layer. The earlier the convolutional layer, the more important the features that are extracted.^[^ ^73^, ^75^ ^]^ Therefore, layers C1 to C4 are sensitive to noise, and it is necessary to reduce the dynamic variation by applying a high *V*_Read_ to these layers to increase the accuracy. Figure 7b shows the accuracy when the selected layer is biased with a low *V*_Read_ (for high read noise), and the other layers are biased with a high *V*_Read_ (for low read noise). The red dotted line indicates the baseline accuracy when a high *V*_Read_ is applied in all layers (89%). Increasing noise in the C1 layer shows dramatic accuracy degradation, resulting in a decrease in accuracy by 7.91%p. The C2 to C5 layers show moderate accuracy degradation (1.35 to 1.11%p), and the C7 to F3 layers show almost no degradation (0.41 to 0.25%p). Therefore, these layers can be used as power‐saving layers with a low *V*_Read_, thereby reducing power consumption without sacrificing accuracy. Moreover, to select the layer that serves as an accuracy‐improving layer and a power‐saving layer, it is important to consider the number of weight parameters in each layer as well as the noise sensitivity. This is because the number of parameters is related to the power consumption of the entire neuromorphic system. If the noise sensitivity is similar between the two layers, it is preferable to choose a layer with fewer parameters to improve accuracy while maintaining low power consumption. The number of parameters in each layer is shown in Figure 7c. The number of parameters of layers C1, C2, F1, F2, and F3 is relatively small. Among them, layers C1 and C2 are the most noise‐sensitive, as shown in Figure 7a. Therefore, it is desirable to apply a high *V*_Read_ to the C1 and C2 layers to improve the accuracy, which we refer to as the accuracy‐improving layers. In contrast, layers C5–C8 have the highest number of parameters. In addition, these layers are noise‐robust, as shown in Figure 7b. Therefore, applying a low *V*_Read_ to these layers can achieve significant power savings while maintaining an acceptable level of accuracy; these layers are called power‐saving layers. In addition, for system stabilization, layers F2 and F3 are designated as accuracy‐improving layers. The rationale underlying this decision is that if the middle layer in the network becomes noisy, the subsequent layers must adapt and counteract this noise. However, if the last layer becomes noisy, the system may become unstable because it may not be able to effectively compensate for the noise. As shown in Figure 7b, when a large noise is introduced to layers F2 and F3, the average decrease in accuracy is small; however, there is a significant variation in the accuracy and presence of outliers, indicating system instability. Therefore, it is preferable to apply a high *V*_Read_ to layers F2 and F3 to ensure their stabilization and overall network performance, given their relatively small number of parameters. Figure 7d shows the overall ARB scheme of the system. Figures 7e,f show the averaged learning accuracy for the conventional bias scheme (*V*_Read_ = 1.75 V, area = 3.53 × 3.53 µm^2^, which shows the lowest power consumption in Figure 6e) and ARB methods (area = 1 × 1 µm^2^). Figure 7g shows the accuracies of the conventional (red) and ARB (green) methods for the 40^th^ (early stage) and 100^th^ (late stage) epochs. The results show that the ARB method has a convergence speed, final accuracy, and stability similar to those of the conventional method. Figure 7h shows the power consumption and area of the ARB and conventional methods. Although the two biasing methods exhibit similar performances (Figure 7g), ARB has 61.3% lower power consumption and 91.9% better density than the conventional method. Therefore, a breakthrough for implementing area‐ and power‐efficient neuromorphic systems is presented in this study. Notably, the proposed ARB method can be applied not only to FTJs, but also to many other devices with 1/*f* noise behavior (ReRAM, PCM, and FET), thus providing a general solution for neuromorphic systems.^[^ ^40^, ^41^, ^42^, ^43^, ^44^, ^45^, ^46^, ^47^, ^48^, ^49^, ^76^, ^77^ ^]^

## 3 Conclusion

We proposed an ARB method to address the performance and scaling limitations in ferroelectric‐based neuromorphic devices, focusing on mitigating the effects of nonideal factors such as static and dynamic variations. Our investigation identified dynamic variation as a key challenge in achieving high accuracy in neuromorphic systems. To address the accuracy degradation caused by the intrinsic noise of the device, we analyzed the LFN of the FTJs. Our results show that the intrinsic noise of FTJs increases with decreasing device size and *V*_Read_, which limits the implementation of both area‐ and power‐efficient neuromorphic systems. To overcome this issue, the ARB method was used, which applies different read biases to each layer of a neuromorphic system. This method takes advantage of the fact that each layer in a neuromorphic system has a different performance sensitivity to the noise of the device, and that noise is related to the read bias. This allows a noise‐robust layer to be used as a power‐saving layer and a noise‐sensitive layer to be used as an accuracy‐improving layer. Our proposed adaptive read biasing method demonstrated 61.3% power savings and 91.9% scaling improvements compared with the conventional biasing method. These results contribute significantly to the development of accurate, efficient, and scalable ferroelectric neuromorphic systems. The ARB method can also be applied to neuromorphic systems using other noisy devices.

## 4 Experimental Section

### Fabrication Process of the HfO2‐FTJ

Figure S1 (Supporting Information) shows the fabrication process of the undoped HfO_2_ FTJ array. 300 nm SiO_2_ was grown on Si substrate via wet oxidation and deposited 100 nm *n* ^+^ polysilicon by low‐pressure chemical vapor deposition (LPCVD) for the bottom electrode of the FTJ. Then, 1.2 nm SiO_2_ was grown by chemical oxidation using an APM solution (H_2_O: H_2_O_2_: NH_4_OH = 5:1:1, 80 °C, 20 min) for the DE layer of FTJ. 6 nm undoped HfO_2_ was deposited by thermal atomic layer deposition (ALD) at 340 °C for the FE of FTJ. Tetrakis (hafnium) was used as the Hf precursor, and ozone was used as the oxidant. Then, 100 nm TiN was deposited by DC sputtering at 200 °C for the top electrode. Photolithography and dry etching were used to define the TiN top gate. To induce ferroelectricity of the HfO_2_ layer, post‐metal annealing (PMA) (N_2_ ambient, 150 sccm, 5 torr) at 800 °C for 30 s was performed. A 300 nm SiO_2_ layer was deposited with tetraethylorthosilicate (TEOS) via LPCVD to form an interlayer dielectric (ILD). After the contact etching of the ILD, metallization was performed with a Ti/TiN/Al/TiN (30/30/300/30 nm) stack.

### Device Spectroscopy Analysis of HfO2‐FTJ

Figure S2 (Supporting Information) shows the material characteristics of the fabricated HfO‐FTJ. Transmission electron microscopy (TEM) images of the fabricated FTJ (TiN/HfO_2_/SiO_2_/Si) showed the thicknesses of the SiO_2_ and HfO_2_ layers. The composition ratio of Hf:O in the HfO_2_ layer was determined by X‐ray photoelectron spectroscopy (XPS) analysis. Grazing incidence X‐ray diffraction (GIXRD) analysis of the as‐deposited HfO_2_ and after PMA was performed. It was confirmed that the PMA at 800 °C crystallized the HfO_2_ to induce the o‐phase, which generated the ferroelectricity.

### Electrical Measurement of the HfO_2_‐FTJ

To measure the electrical characteristics of the HfO_2_‐FTJ devices, a semiconductor parameter analyzer (B1500A, Keysight) and a waveform generator/fast measurement unit (WGFMU) module of the B1500 (Keysight) were used.

Figure S7 (Supporting Information) shows the measurement setup for LFN spectroscopy, where a low‐noise amplifier (SR570) and signal analyzer (35670A) are utilized to measure the LFN of the FTJ. The output current of the HfO_2_‐FTJ was connected to SR570, which converted the current fluctuation into a low‐noise voltage fluctuation. The voltage fluctuation was converted to the power spectral density at 35670A. The floor of the noise level was lower than 10^−23^ A^2^ Hz^−1^, which showed that the current noise of the FTJ measured in the subsequent experiments was a reliable result.

### Software‐Based Simulation

Simulations for the classification of CIFAR‐10 were performed using Python software. The nonideal characteristics of the FTJ, such as nonlinearity, asymmetry, and variation, were reflected in the simulation. The power consumption during learning and inference was calculated using Python.

### Calculation of Power Consumption

The power consumption of individual cells was calculated using the formula.^[^ ^78^ ^]^

(8) $$E_{\mathrm{cell}}=V_{\mathrm{Read}}^{2}\times G_{\mathrm{cell}}\times T_{\mathrm{read}}$$

where *E*_cell_ is the energy consumption of a single cell, *G*_cell_ is the conductance of the cell, and *T*_read_ is the width of the read pulse. By summing the energy consumed by all cells, the overall energy consumption of the array in the neuromorphic system could be calculated. Furthermore, the neuron circuit's operational energy was estimated by the following equation.^[^ ^78^ ^]^

(9) $$E_{\mathrm{TIA}}=n_{\mathrm{neuron}}\times T_{\mathrm{read}}\times 0.34\mathrm{mW}$$

where *E*_TIA_ is the energy consumption of neurons, and *n*_neuron_ is the number of neuron circuits. By adding *E*_cell_ and *E*_TIA_, the synaptic array's total power consumption was calculated.

## Conflict of Interest

The authors declare no conflict of interest.

## Supporting information

Supporting information

## Data Availability

The data that support the findings of this study are available from the corresponding author upon reasonable request.
