# Supplementary material for: Proposition of Adaptive Read Bias: A Solution to Overcome Power and Scaling Limitations in Ferroelectric‐Based Neuromorphic System
Source: Adv Sci (Weinh). 2023 Dec 1;11(5):2303735. doi: 10.1002/advs.202303735 (PMC10837350; doi:10.1002/advs.202303735)
Supplement: Supplementary file 1 — Supporting information [file ADVS-11-2303735-s001.pdf]

ADVANCED SCIENCE
Open Access

Supporting Information

for *Adv. Sci.*, DOI 10.1002/advs.202303735

Proposition of Adaptive Read Bias: A Solution to Overcome Power and Scaling Limitations in Ferroelectric-Based Neuromorphic System

Ryun-Han Koo, Wonjun Shin, Seungwhan Kim, Jiseong Im, Sung-Ho Park, Jong Hyun Ko, Dongseok Kwon, Jae-Joon Kim, Daewoong Kwon and Jong-Ho Lee**

Supporting Information

Proposition of Adaptive Read Bias: A Solution to Overcome Power and Scaling Limitations in Ferroelectric-based Neuromorphic System

Ryun-Han Koo[1], Wonjun Shin[1], Seungwhan Kim[1], Jiseong Im[1], Sung-Ho Park[1], Jong Hyun Ko[1], Dongseok Kwon[1], Jae-Joon Kim[1], Daewoong Kwon[2*], and Jong-Ho Lee[1*,3]

[1]*Inter-University Semiconductor Research Center, Department of Electrical and Computer Engineering, Seoul National University, Seoul 08826, Korea*

[2]*Department of Electrical Engineering, Hanyang University, Seoul 04763, Korea*

[3]*Minstry of Science and ICT, Sejong, Korea*

Ryun-Han Koo and Wonjun Shin contributed equally to this work.

*corresponding author: Daewoong Kwon and Jong-Ho Lee

Supplementary text

1. Current conduction mechanism of HfO_2-FTJ in HRS.

We have demonstrated that the current conduction mechanism of HfO_2-FTJ in the high-resistance state (HRS) follows trap-assisted direct tunneling using two different methods. First, Figure S6(a) shows the relationship between current density (J) and inverse of electric field (E^{-1}) as a function of temperature (T) in HfO_2-FTJ in the HRS. Over all temperatures, the region from E^{-1} 0 to 1 $(MV/cm)^{-1}$ (highlighted by a pink ellipse) exhibits a linear fit, indicating current conduction by trap-assisted direct tunneling. Second, we used LFN spectroscopy to accurately distinguish the current conduction mechanism. LFN spectroscopy is a valuable tool for identifying carrier transport mechanisms due to its high sensitivity to interface quality, trapping/detrapping time constants, and mobility fluctuations. Figure 6(b-1) shows the dependence of normalized current power spectral density (S_{IT}/I_T^2) on frequency (f) with increasing read voltage (V_{Read}). At the lowest V_{Read} (1.5 V), shot noise is observed for f above 100 Hz. At V_{Read} above 2.0 V, shot noise is completely absent in the frequency range from 10 to 1600 Hz, and only 1/f noise is observed. This phenomenon is known to result from trap-assisted tunneling [S1]. At low V_{Read}, the wide f-range where shot noise dominates is due to a large Fano factor (F) caused by the longer trapping time constant of Si (τ_{Si}). As V_{Read} increases, τ_{Si} decreases, resulting in a smaller F and a tendency toward 1/f noise due to barrier height fluctuations across the SiO_2-Si interface as shown in Figure S6(b). Based on these two findings, we can conclude that the current conduction mechanism of the HfO_2-FTJ in the HRS is trap-assisted direct tunneling [S2].

2. Current conduction mechanism of HfO_2-FTJ in LRS

We have demonstrated that the current conduction mechanism of HfO_2-FTJ in the low-resistance state (LRS) follows the Poole-Frenkel (PF) emission using two different methods. First, Figure S7(a) shows the relationship between J/E and $E^{1/2}$ as a parameter of T in HfO_2-FTJ in the LRS. It shows a linear fit over all temperatures. This indicates current conduction by PF emission. For further demonstration, the trap energy level (φ_t) of the PF emission is extracted. Figure S7(b) and (c) show the process of extracting φ_t. The extracted φ_t is 0.223 eV, which is consistent with the trap generated by interstitial hydrogen atoms, which is a common phenomenon for HfO_2 layer [S3].

Second, we used LFN spectroscopy to accurately distinguish the current conduction mechanism. Figure 6(c-1) shows the dependence of S_{IT}/I_T^2 on f with increasing V_{Read}. As V_{Read} increases, S_{IT}/I_T^2 decreases. When the conduction mechanism follows Fowler-Nordhiem (FN) tunneling, S_{IT}/I_T^2 remains constant regardless of V_{Read}. The tendency of S_{IT}/I_T^2 to decrease with increasing V_{Read} indicates that the current conduction mechanism is PF emission and not FN tunneling [S2]. Based on these two findings, we can conclude that the current conduction mechanism of the HfO_2-FTJ in the LRS is PF emission.

3. Adaptive read bias method with random telegraph noise

This study primarily addresses 1/*f* noise, which is the most prevalent form of noise in semiconductor devices. However, if carrier transport involves only a limited number of traps, such as in very small devices, the current can exhibit intermittent transitions between two or more discrete states—a phenomenon known as random telegraph noise (RTN) [S4, S5]. Therefore, it is important to demonstrate that the proposed ARB method remains applicable and effective even in the presence of RTN.

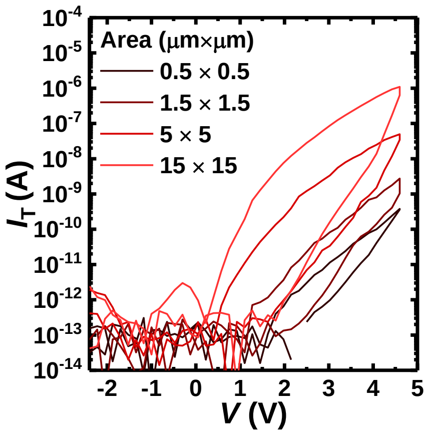

Figure ST 3-1. Double sweep I_T versus V as a parameter of area.

We experimentally confirm whether a reduction in noise magnitude in devices with RTN is indeed observed as V_{Read} increases. Since the HfO_2 FTJs used in this paper exhibited 1/*f* noise, we fabricated FTJs with different fabrication parameters of HfO_2. Figure ST 3-1 shows the I_T versus V of this FTJ as a parameter of area. Figure ST 3-2 shows the I_T values sampled at 3200 Hz for 5 seconds at various V_{Read} values: 3.0 (a), 3.3 (b), 3.6 (c), and 3.9 (d). An RTN is observed at V_{Read} of 3.0, 3.3, and 3.6 V, with a decreasing magnitude as the V_{Read} increases. At 3.9 V, the RTN disappears completely. Figure ST 3-3 (a) shows the correlation between normalized current fluctuation and V_{Read}. The normalized current fluctuation decreases as V_{Read} increases as the number of carriers increases. Figure ST 3-3 (b) shows S_{IT}/I_T^2 versus frequency as a parameter of V_{Read}. At a V_{Read} of 3.0 V and 3.6 V, RTN arises, causing $1/f^2$ noise, while at 3.9 V V_{Read}, 1/*f* noise arises without RTN. Furthermore, as V_{Read} increases, S_{IT}/I_T^2 decreases. The experimental results show that the magnitude of $1/f^2$ noise decreases with increasing V_{Read}. As the ARB method relies on the principle that noise magnitude decreases with higher V_{Read} values, our findings affirm the applicability of ARB to electrical devices that exhibit RTN.

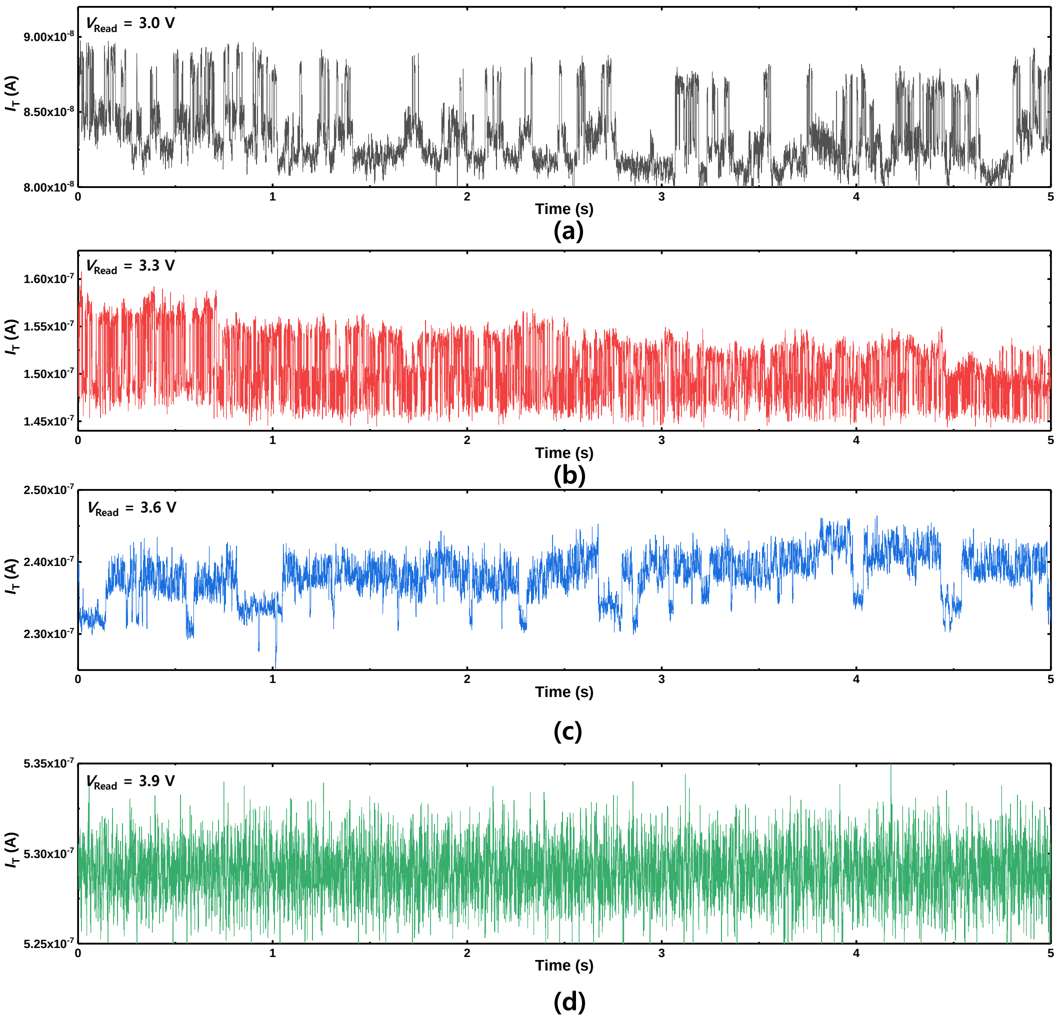

Figure ST 3-2. The I_T values sampled at 3200 Hz for 5 seconds at various V_{Read} values: 3.0 (a), 3.3 (b), 3.6 (c), and 3.9 (d).

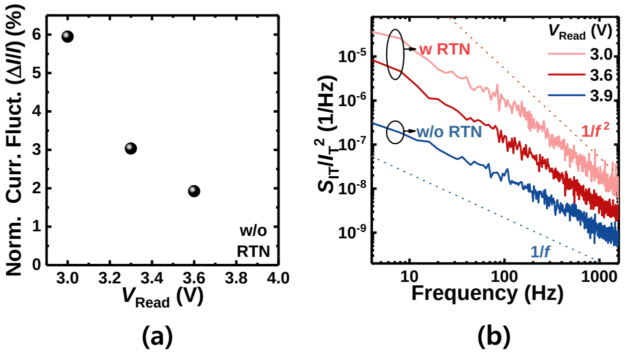

Figure ST 3-3. (a) shows the correlation between normalized current fluctuation and V_{Read}. (b) S_{IT}/I_T^2 versus frequency as a parameter of V_{Read}.

In addition, we investigate how RTN changes in the presence of cycling stress. Figures ST 3-4 (a) and (b) show the I_T values sampled at 3200 Hz for 5 seconds in pristine and damaged devices, respectively. For damaged devices, a 10^5 endurance cycling pulse was applied. On the damaged devices, we observed that the RTN disappeared. Figure ST 3-4 (b) shows the S_{IT}/I_T^2 versus frequency in both pristine and damaged devices. The pristine device exhibits $1/f^2$ noise, whereas the damaged device exhibits $1/f$ noise. Moreover, the amplitude of the noise is greater for the damaged device. In a pristine device, a single trap acts as a dominant noise source, resulting in $1/f^2$ noise. However, when cycling stress is applied, the number of traps that act as noise sources during the carrier transport mechanism increases, resulting in multiple $1/f^2$ noise sources with different time constants. These additional noise sources superimpose upon the original RTN, ultimately giving rise to the manifestation of $1/f$ noise. Additional measurements were performed to demonstrate that ARB is applicable to damaged devices. Figure ST 3-4 (c) shows the relationship between S_{IT}/I_T^2 and frequency as a function of V_{Read} in a damaged device. Even for damaged devices, we were able to experimentally confirm that S_{IT}/I_T^2 decreases as V_{Read} increases. Thus, it shows that adaptive read bias can still be applied to damaged devices.

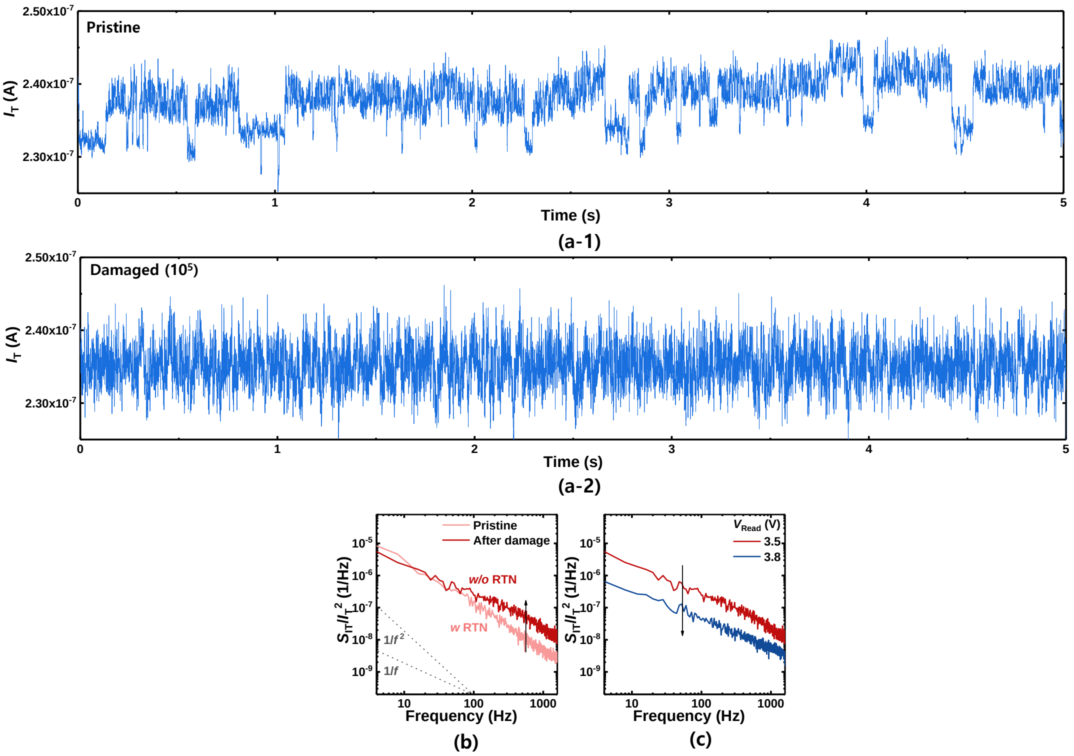

Figure ST 3-4. I_T values sampled at 3200 Hz for 5 seconds in (a-1) pristine and (a-2) damaged devices. (b) S_{IT}/I_T^2 versus frequency in both pristine and damaged devices. (c) The relationship between S_{IT}/I_T^2 and frequency as a function of V_{Read} in a damaged device.

4. The size of PSD investigated devices

Table S1 contains detailed information on the measurement conditions for devices whose PSD has been measured, including their resistance states and measuring bias conditions. The fabricated devices exhibit a size range spanning from 100 × 100 μm^2 to 0.5 × 0.5 μm^2. Figure ST 4-1 shows double sweep I_T versus V as a parameter of area. However, it is important to note that PSD measurements cannot be obtained for all these devices. In our measurement setup, which is consistent with the approach commonly employed in PSD measurement systems, the current is amplified using a current preamplifier (SR570). In this case, it is essential that the read current (I_T) exceeds 1 nA to ensure the reliability of PSD measurements, given the inherent limitations in the bandwidth of the SR570. Therefore, we extrapolate the PSD values for the smaller device whose I_T is smaller than 1 nA.

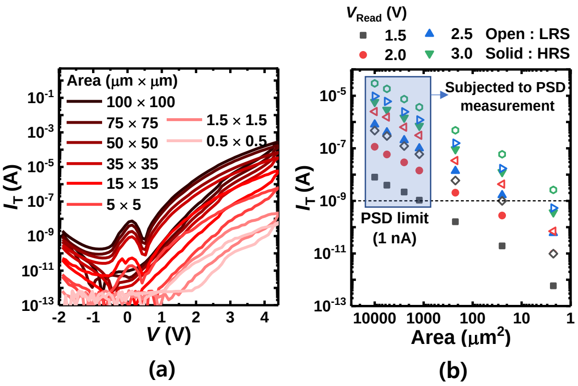

Figure ST 4-1. (a) Double sweep I_T versus V as a parameter of area. (b) I_T versus area as a parameter of V_{Read}. blue box denotes that the area of FTJ needs to be greater than 35 × 35 μm^2 to ensure reliable PSD measurement.

In the manuscript, the PSD is measured on devices larger than 35 × 35 μm^2. This criterion was established because the area of FTJ needs to be greater than 35 × 35 μm^2 to ensure reliable PSD measurement in both LRS and HRS at the V_{Read} values ranging from 1.5 V to 3.0 V (Figure ST 4-1(b)). In supplementary section, we expanded our PSD measurements to encompass devices as small as 1.5 × 1.5 μm^2 by constraining the measuring bias range. Down till a device size of 5 × 5 μm^2, the PSDs are measured for both LRS and HRS at a V_{Read} of 3.0 V. Down till the device size 1.5 × 1.5 μm^2, we focused solely on PSD measurements in LRS at a read bias of 3.0 V. The result of PSD measurements for FTJ measuring from 100 × 100 μm^2 to 5.0 × 5.0 μm^2 at V_{Read} = 3.0 V are shown in Figure ST 4-2 (a). Both LRS (Figure ST 4-2(a-1)) and HRS (Figure ST 4-2(a-2)) exhibit a linear increase in S_{IT}/I_T^2 as the area decreases. Figure ST 4-2 (b) shows the relationship between S_{IT}/I_T^2 and the area of FTJ. In both HRS and LRS, the S_{IT}/I_T^2 values are inversely proportional to the area, thus confirming the validity of the extrapolation method employed in this study.

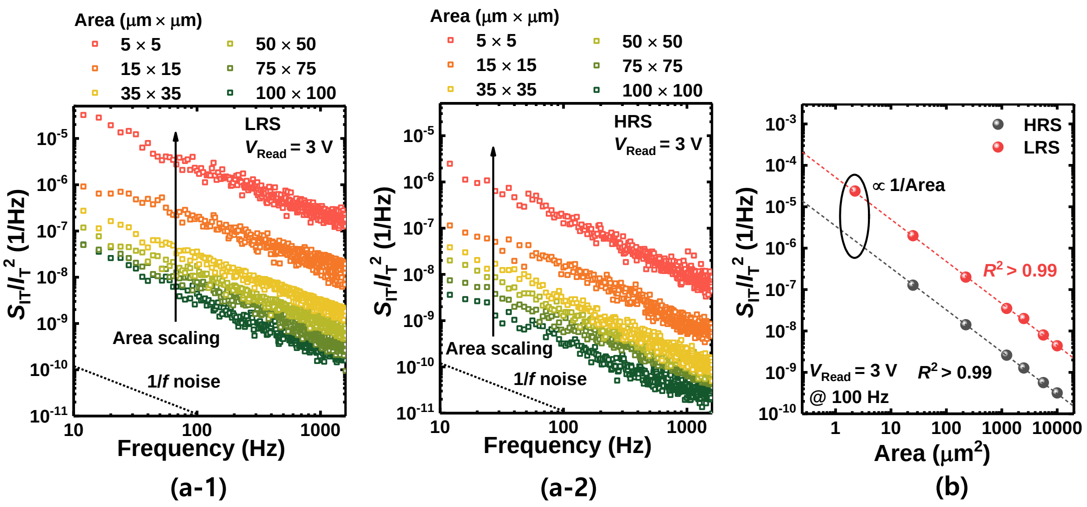

Figure ST 4-2. S_{IT}/I_T^2 versus f as a parameter of the device area in LRS (a-1) and HRS (a-2). (b) S_{IT}/I_T^2 with respect to the device area in HRS and LRS.

Supplementary figures

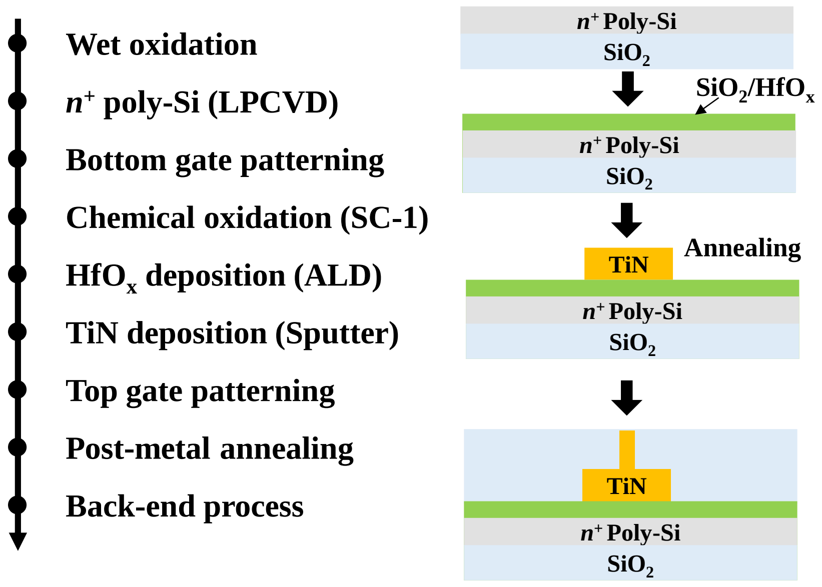

Figure S1. Fabrication process for HfO_2 FTJ. 300 nm SiO_2 is grown by wet oxidation on Si substrate and 100 nm n^+ polysilicon is deposited by low pressure chemical vapor deposition (LPCVD). 1.2 nm SiO_2 for DE of FTJ grown by chemical oxidation using APM solution (H_2O : H_2O_2 : NH_4OH = 5 : 1 : 1, 80 ℃, 20 min). 6 nm HfO_2 for FE of FTJ deposited by thermal atomic layer deposition (ALD) at 340 ℃. 100 nm TiN for top gate deposited by DC sputtering at 200 ℃. Photolithography and dry etching are performed to define the TiN top gate. After define top gate, post-metal annealing (PMA) performs at 800 ℃, 30 s with N_2 ambient to induce ferroelectricity of HfO_2 layer. A 300 nm SiO_2 deposited with tetraethylorthosilicate (TEOS) by LPCVD to form the interlayer dielectric (ILD). After contact etching of the ILD, metallization is performed with Ti/TiN/Al/TiN (30 nm/30 nm/ 300 nm/30 nm) stack.

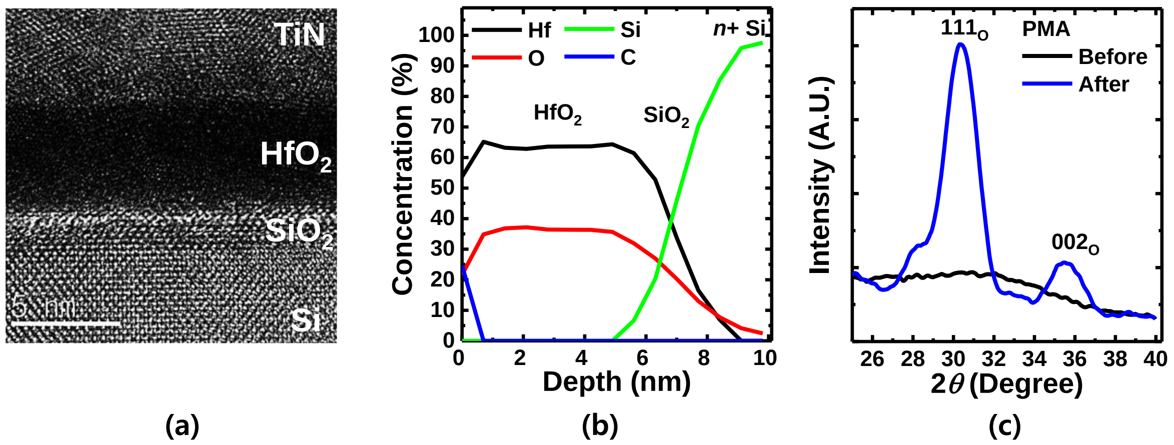

Figure S2. Material characteristics of fabricated HfO_2-FTJ. a) A transmission electron microscopy (TEM) image of the fabricated FTJ (TiN/HfO_2/SiO_2/Si). b) Composition ratio of HfO_2 layer is analyzed through X-ray photoelectron spectroscopy (XPS). (c) The crystal structure of the HfO_2 layer is examined using grazing incidence x-ray diffraction (GIXRD).

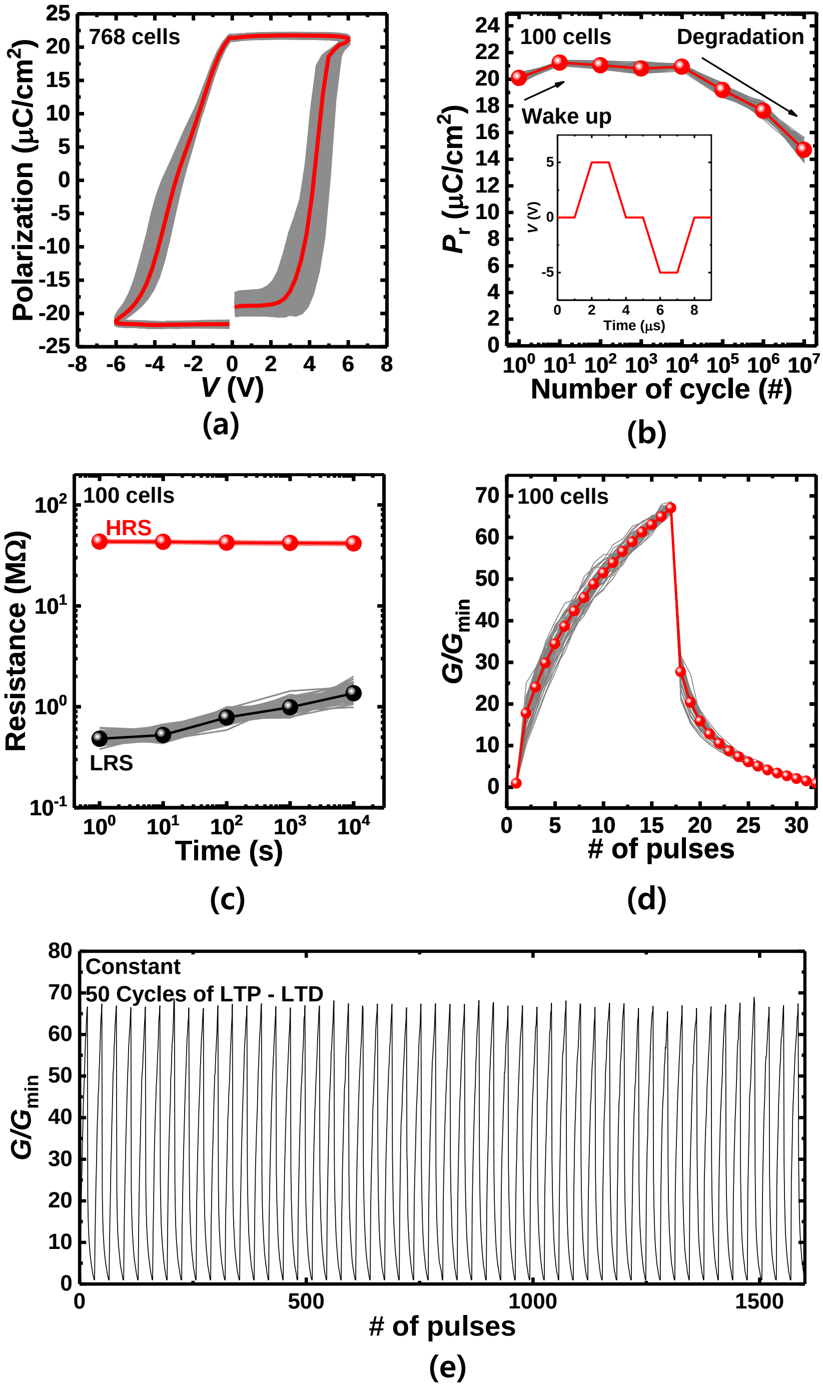

Figure S3. (a) *P-V* curves of the 768 HfO_2-FTJ cells in 32 × 24 array (gray lines) and averaged *P-V* curves (red line). (b) Endurance characteristics of the 100 HfO_2-FTJ cells (gray lines) and averaged endurance characteristic (red line). Inset shows pulse used for cycling stress. (c) Retention characteristics of the 100 HfO_2-FTJ cells cells (lines) and their averaged retention characteristics (lines with symbol). (d) LTP-LTD characteristics of the 100 HfO_2-FTJ cells (gray lines) and averaged LTP-LTD characteristics (red line) with an identical pulse scheme. An identical pulse of 5 V, 1 μs is applied. (e) 50 repetitive LTP-LTD characteristics of fabricated HfO_2-FTJ with identical pulse scheme. During a total of 1600 spikes of presynaptic pulses, HfO_2-FTJ shows stable operation, exhibiting excellent cycle to cycle variation.

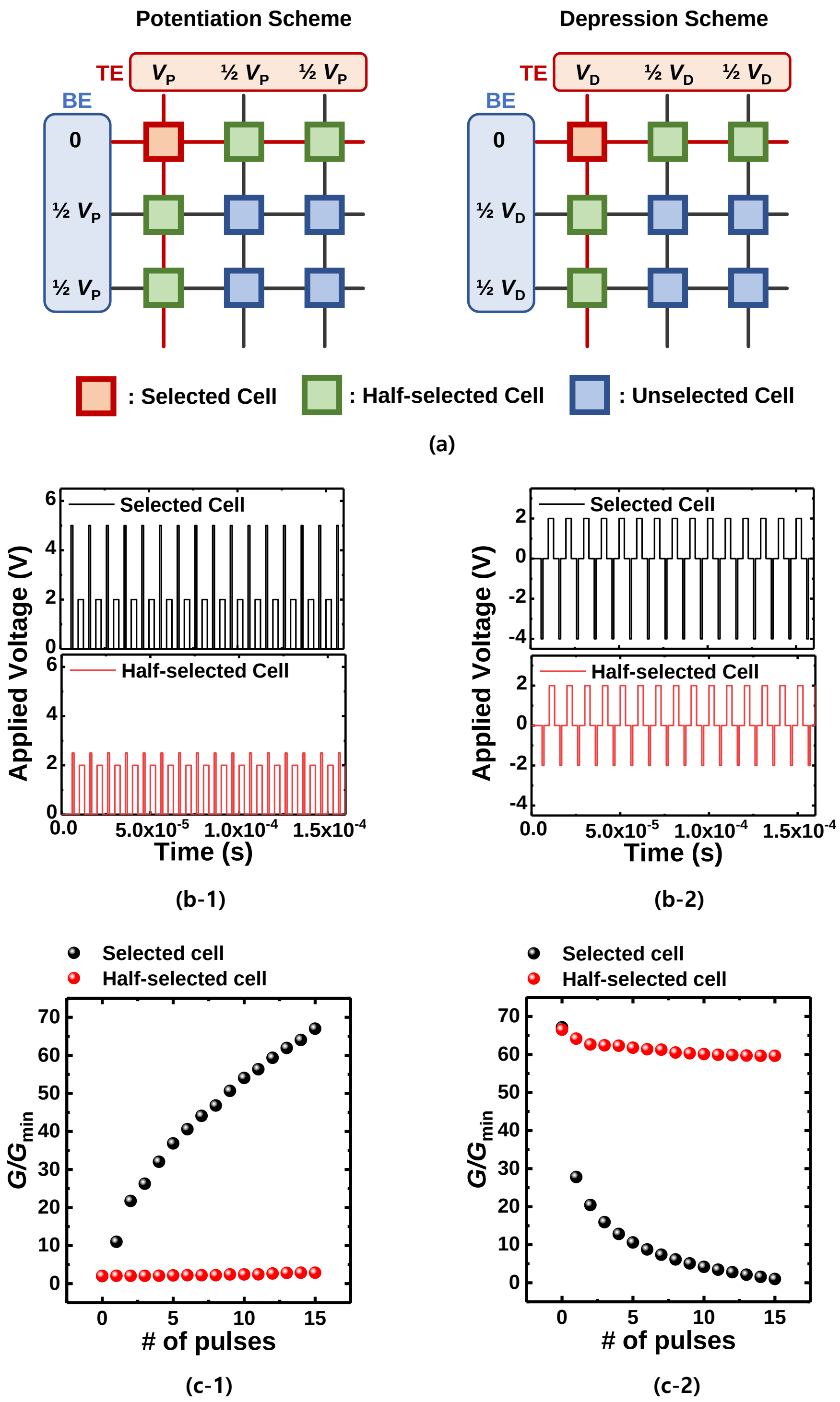

Figure S4. (a) Schematic diagram of potentiation and depression of the HfO_2-FTJ array. Voltage applied to the selected cell and half-selected cells during potentiation (b-1) and depression (b-2). The conductance change of selected and half-selected cells during potentiation (c-1) and depression (c-2).

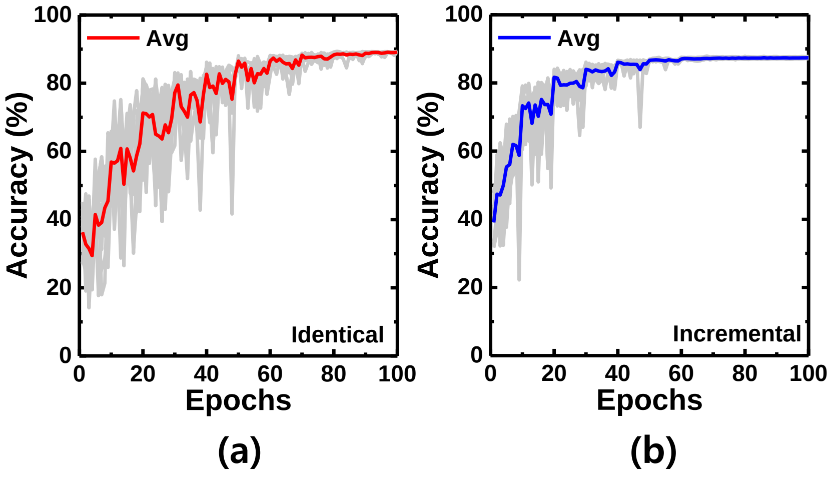

Figure S5. Comparison of learning accuracy between identical and incremental pulse schemes. a) Averaged learning accuracy (red line) for 10 learning curves (gray lines) using an identical pulse scheme. b) Averaged learning accuracy (blue line) for 10 learning curves (gray lines) using an incremental pulse scheme.

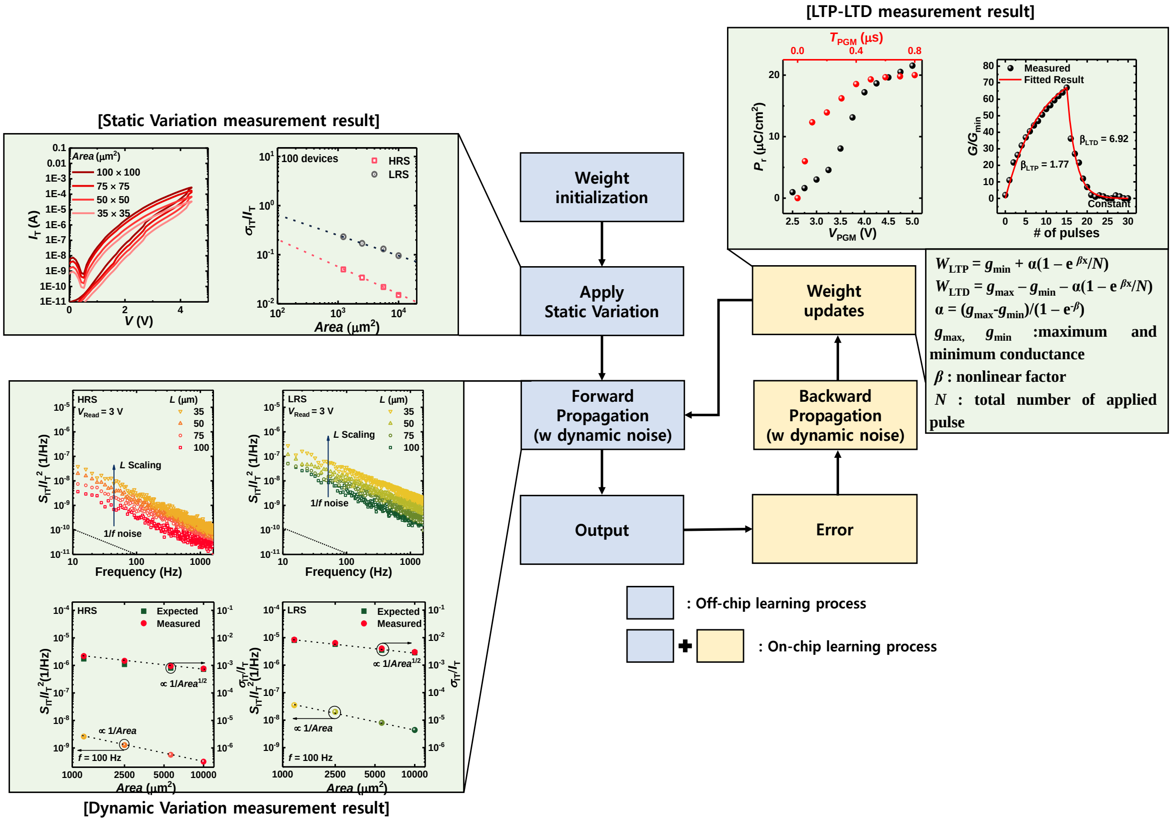

Figure S6. Schematic diagram of simulation methodology. After weight initialization, a static variation is applied by multiplying random normally distributed matrixes to weight matrix (W), minimum conductance (g_{min}), and maximum conductance (g_{max}) of each cells. During forward propagation process, dynamic noise affects the read current. In on-chip learning process, the difference between the classification result and the error is used for backpropagation. When updating as a result of backpropagation, LTP-LTD nonlinearity factors are reflected.

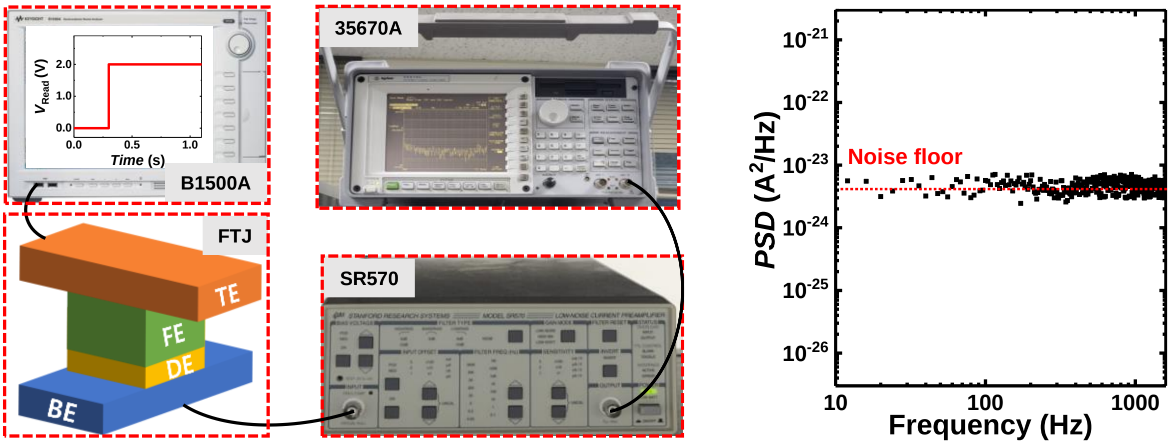

(a) (b)

Figure S7. Measurement setup for LFN spectroscopy. a) A semiconductor parameter analyzer (B1500A, Keysight) is used to apply the V_{Read} bias to the TiN top electrode. The output current of the HfO2-FTJ is connected to a low noise current amplifier (SR570). SR570 converts output current fluctuation into low-noise voltage fluctuation. Voltage fluctuation is connected to signal analyzer (35670A) and converted to power spectral density. b) Noise floor of measurement setup is lower than 10^{-23} A^2/Hz, which shows that the current noise of FTJ measured in subsequent experiments is a reliable result.

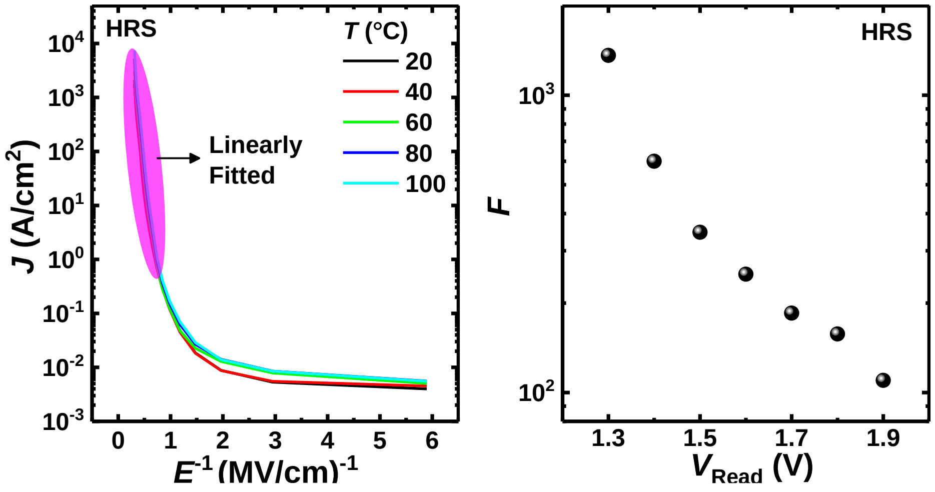

(a) (b)

Figure S8. Analysis of the current conduction mechanism of HfO_2-FTJ in high-resistance state (HRS). a) J versus E^{-1} at different T (20, 40, 60, 80, and 100 °C). The linearly fitted region shows the current conduction mechanism of HfO_2-FTJ in HRS following trap-assisted direct tunneling. b) Fano factor (F) versus V_{Read}. As V_{Read} increases, the shot noise decreases and $1/f$ noise appears. These two results show that the current conduction mechanism follows trap-assisted direct tunneling.

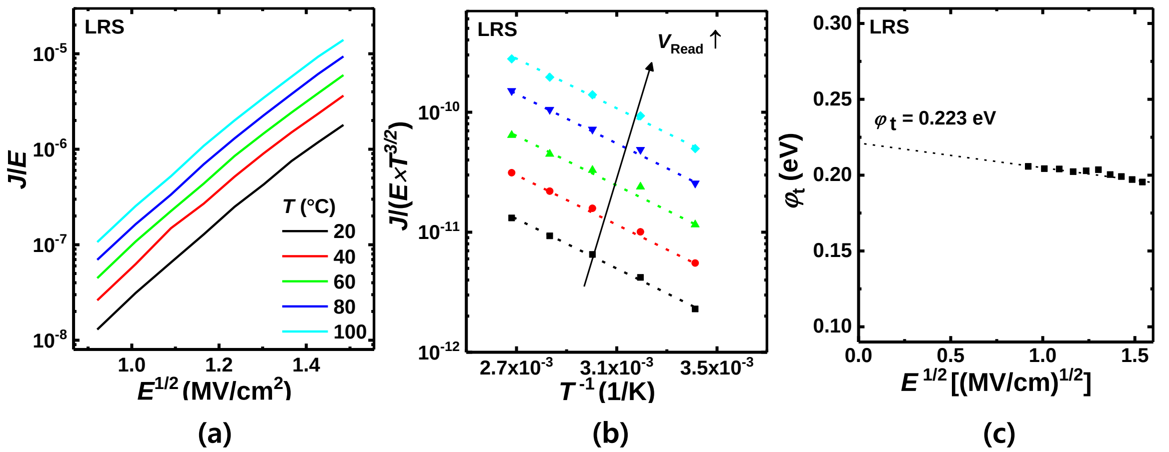

Figure S9. Analysis of current conduction mechanism of HfO_2-FTJ in low resistance state (LRS). a) J/E versus $E^{1/2}$ at different T (20, 40, 60, 80, 100 °C) in HfO_2-FTJ in LRS. b) $J/(E \times T^{3/2})$ versus T^{-1} at different V_{Read} in HfO_2-FTJ in LRS. c) Extracted trap energy level (φ_t) from the slope of b). φ_t of the HfO_2 layer is 0.223 eV, which is consistent with the trap level generated by intersitial hydrogen atoms.

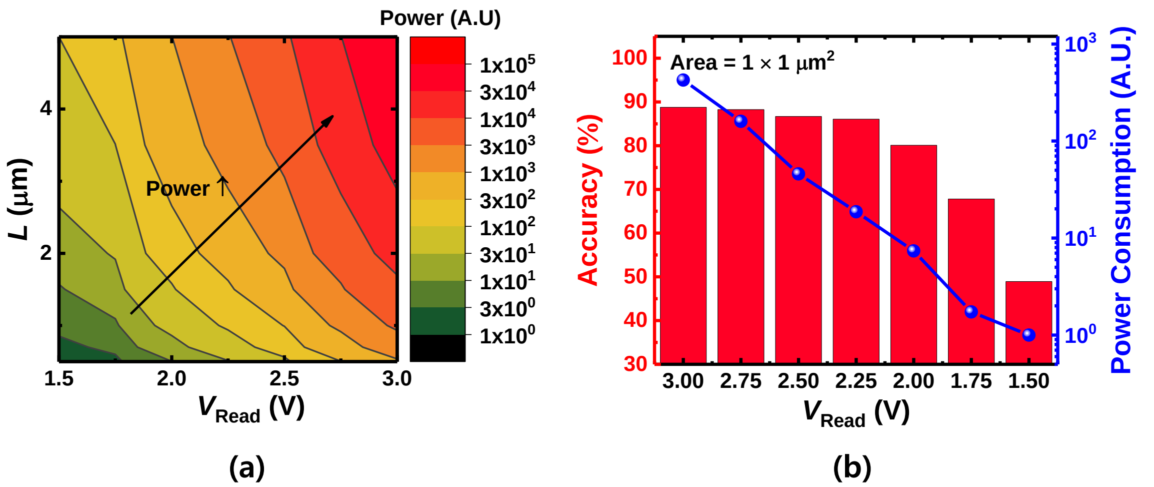

Figure S10. Relationship between area, V_{Read}, power and accuracy in HfO_2-FTJ based neuromorphic system considering dynamic variation of the device. a) Power consumption of HfO_2-FTJ based neuromorphic system with respect to change of L and V_{Read}. As the area and V_{Read} of the hardware neuromorphic system increase, the power increases exponentially. b) Accuracy (red axis) and power consumption (blue axis) of the HfO_2-FTJ based neuromorphic system with a fixed area of $1 \times 1\ \mu m^2$ with different V_{Read} bias of the system. As the V_{Read} decreases, the accuracy of the system also decreases, indicating a major bottleneck in the implementation of a low-power, high-density, and high-accuracy system.

Supplementary table

Area (μm × μm)	Fabricated & *I*-*V* measured devices	PSD investigated devices		Theoretically studied devices
		LRS	HRS	
**100 × 100**	**O**	V_{Read} **= 1.5 to 3.0 V**	V_{Read} **= 1.5 to 3.0 V**	
**75 × 75**	**O**	V_{Read} **= 1.5 to 3.0 V**	V_{Read} **= 1.5 to 3.0 V**	
**50 × 50**	**O**	V_{Read} **= 1.5 to 3.0 V**	V_{Read} **= 1.5 to 3.0 V**	
**35 × 35**	**O**	V_{Read} **= 1.5 to 3.0 V**	V_{Read} **= 1.5 to 3.0 V**	
**17× 17**	**-**			**Interpolation**
**15 × 15**	**O**	V_{Read} **= 1.5 to 3.0 V**	V_{Read} **= 2.0 to 3.0 V**	
**5.29 × 5.29**	**-**			**Extrapolation**
**5 × 5**	**O**	V_{Read} **= 1.5 to 3.0 V**	V_{Read} **= 2.5 to 3.0 V**	
**1.5 × 1.5**	**O**	V_{Read} **= 3.0 V**		
**0.59 × 0.59**	**-**			**Extrapolation**
**0.5 × 0.5**	**O**			

Table S1. The area of the fabricated device, the area of the device where PSD measurements are performed, and the area of theoretically studied device.

References

[S1] J. Lee, G. Bosman, K. R. Green, D. Ladwig, IEEE Trans. Electron Devices, 2003, 50, 12.
[S2] R. H. Koo, W. Shin, K. K. Min, D. Kwon, D. H. Kim, J. J. Kim, J. H. Lee, IEEE Electron Device Lett, 2022, 44, 1.
[S3] P. W. Peacock, J. Robertson, Appl. Phys. Lett, 2003, 83, 10.
[S4] F. N. Hooge, 1994, 41, 11, 1926-1935.
[S5] M. Haartman, and M. Östling, M. Low-frequency noise in advanced MOS devices. Springer Science & Business Media. 2007.
